# Systemic paralogy and function of retinal determination network homologs in arachnids

**DOI:** 10.1186/s12864-020-07149-x

**Published:** 2020-11-23

**Authors:** Guilherme Gainett, Jesús A. Ballesteros, Charlotte R. Kanzler, Jakob T. Zehms, John M. Zern, Shlomi Aharon, Efrat Gavish-Regev, Prashant P. Sharma

**Affiliations:** 1 Department of Integrative Biology, University of Wisconsin-Madison, Madison, WI 53706 USA; 2 National Natural History Collections, The Hebrew University of Jerusalem , Jerusalem, 9190401, Israel

**Keywords:** Cave blindness, *sine oculis*, *Six1*, *Parasteatoda tepidariorum*, Amblypygi, RNAi

## Abstract

**Background:**

Arachnids are important components of cave ecosystems and display many examples of troglomorphisms, such as blindness, depigmentation, and elongate appendages. Little is known about how the eyes of arachnids are specified genetically, let alone the mechanisms for eye reduction and loss in troglomorphic arachnids. Additionally, duplication of Retinal Determination Gene Network (RDGN) homologs in spiders has convoluted functional inferences extrapolated from single-copy homologs in pancrustacean models.

**Results:**

We investigated a sister species pair of Israeli cave whip spiders, *Charinus ioanniticus* and *C. israelensis* (Arachnopulmonata, Amblypygi), of which one species has reduced eyes. We generated embryonic transcriptomes for both Amblypygi species, and discovered that several RDGN homologs exhibit duplications. We show that duplication of RDGN homologs is systemic across arachnopulmonates (arachnid orders that bear book lungs), rather than being a spider-specific phenomenon. A differential gene expression (DGE) analysis comparing the expression of RDGN genes in field-collected embryos of both species identified candidate RDGN genes involved in the formation and reduction of eyes in whip spiders. To ground bioinformatic inference of expression patterns with functional experiments, we interrogated the function of three candidate RDGN genes identified from DGE using RNAi in the spider *Parasteatoda tepidariorum*. We provide functional evidence that one of these paralogs, *sine oculis/Six1 A* (*soA*), is necessary for the development of all arachnid eye types.

**Conclusions:**

Our work establishes a foundation to investigate the genetics of troglomorphic adaptations in cave arachnids, and links differential gene expression to an arthropod eye phenotype for the first time outside of Pancrustacea. Our results support the conservation of at least one RDGN component across Arthropoda and provide a framework for identifying the role of gene duplications in generating arachnid eye diversity.

## Background

Cave habitats offer apt systems for investigating the genetic basis of morphological convergence because communities of these habitats are similarly shaped by environmental pressures, such as absence of light and diminished primary productivity [1, 2]. Troglobites, species exclusive to cave environments and adapted to life in the dark, exhibit a suite of characteristics common to cave systems around the world, such as reduction or complete loss of eyes, depigmentation, elongation of appendages and sensory structures, and decreased metabolic activity [3–5]. Previous work has shown that troglomorphism can evolve over short time spans (< 50 kyr) despite gene flow [6–8] and that parallel evolution of troglomorphic traits (e.g., depigmentation; eye loss) in independent populations can involve the same genetic locus [9–11].

Troglomorphism and troglobitic fauna have been analyzed across numerous taxonomic groups with respect to systematics and population genetics. However, one component of the troglobitic fauna that remains poorly understood is cave arachnids. Most species of Arachnida are prone to nocturnal habits and some orders broadly exhibit troglophily; in fact, troglobitic species are known from all the extant terrestrial arachnid orders except Solifugae and Thelyphonida [12–20]. In addition to eye and pigment loss, troglomorphism in arachnids manifests in the form of compensatory elongation of walking legs and palps, appendages which harbor sensory structures in this group [21–24].

Thorough understanding of the developmental genetic basis for the evolution of troglomorphic traits has been largely spearheaded in two model systems: the Mexican cave fish *Astyanax mexicanus* [5–8, 10, 25] and the cave isopod *Asellus aquaticus* [4, 9, 26]. Both model systems have more than one hypogean population, can be maintained in the laboratory, and are amenable to approaches such as genetic crosses and quantitative trait locus mapping. The advent of short read sequencing technology in tandem with experimental approaches has transformed the potential to triangulate regulatory differences between hypogean (subterranean) and epigean (surface-dwelling) lineages [3, 9, 10, 26], and to study a broader range of cave taxa. Among arthropods, work on the isopod *A. aquaticus* in particular has made significant advances in the identification of loci regulating pigmentation and size of arthropod eyes [9, 11], complementing forward and reverse genetic screening approaches in other pancrustacean models (e.g., *Drosophila melanogaster*, *Tribolium castaneum*, and *Gryllus bimaculatus*) [27–30]. However, developmental and genetic insights into the evolution of blindness illuminated by *A. aquaticus* and other pancrustacean models are not directly transferable to Arachnida for two reasons. First, the eyes of arachnids are structurally and functionally different from those of pancrustaceans. Typically, the main eyes of adult Pancrustacea (e.g., *A. aquaticus*) are a pair of faceted (or apposition) eyes, which are composed of many subunits of ommatidia. In addition, adult Pancrustacea have small median ocelli (typically three in holometabolous insects), often located medially and at the top of the head.

**Fig. 1:**
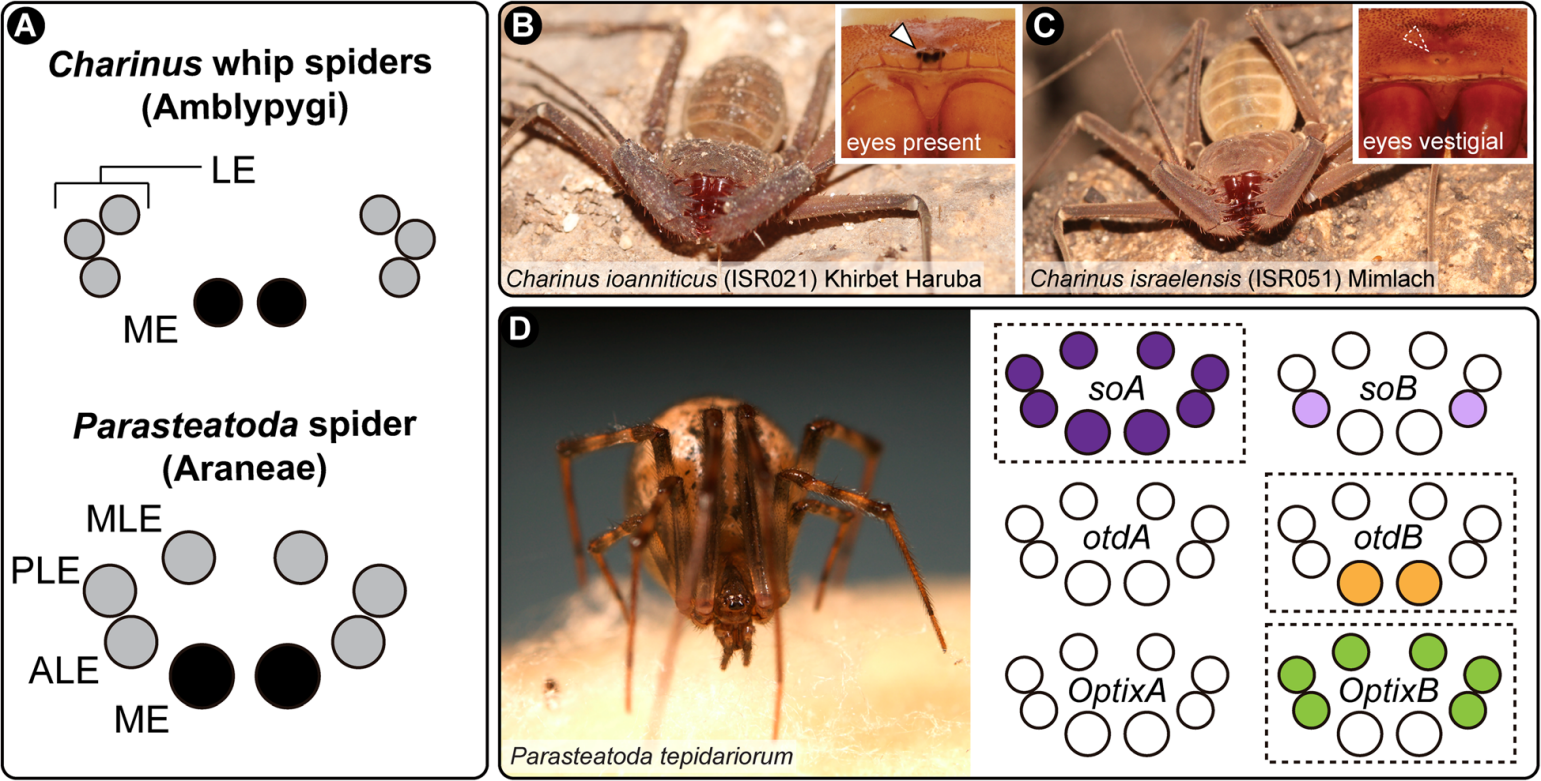
Study species and their corresponding eye arrangements. **a**: Schematic representation of the eyes of *Charinus* whip spiders (Amblypygi) (upper), and the spider *Parasteatoda tepidariorum* (Araneae; lower). **b**: Live specimen of *C. ioanniticus* from Khirbet Haruba cave (Haruva cave). Inset: detail of the median eyes. **c**: Live specimen of *C. israelensis* from Mimlach cave. Inset: detail of the reduced median eyes. **d**: Live specimen of *P. tepidariorum*, and schematic representation of the expression patterns of paralog pairs of *Ptep-sine oculis* (*soA*/*soB*), *Ptep-orthodenticle* (*otdA*/*otdB*), and *Ptep-Optix* (*OptixA*/*OptixB*) in the eyes. ME: median eyes; ALE: anterior lateral eyes; PLE: posterior lateral eyes; MLE: median lateral eyes; LE: lateral eyes. B–C by Shlomi Aharon; D by Jesús A. Ballesteros

By contrast, extant arachnids lack ommatidia and typically have multiple pairs of eyes arranged along the frontal carapace. All arachnid eyes are simple-lens eyes or ocelli; each eye has a single cuticular lens, below which are a vitreous body and visual cells. The retina is composed of the visual cells and pigment cells. These eyes are divided in two types, namely the principal eyes and the secondary eyes [31, 32]. Principal and secondary eyes differ in the orientation of their retina [33]: the principal eyes are of the everted type, with the visual cells lying distally, and lack a reflective layer; the secondary eyes are inverted, with the light-sensitive rhabdomeres pointing away from incoming light (analogous to vertebrate eyes). All secondary eyes possess a reflective layer of crystalline deposits called a tapetum, which is responsible for the “eye shine” of spiders. The principal eyes are the median eyes (ME, also known as anterior median eyes). The secondary eyes comprise the anterior lateral eyes (ALE), posterior lateral eyes (PLE), and median lateral eyes (MLE; also known as posterior median eyes) (Fig. 1a) [31, 32] (nomenclature used here follows Schomburg et al. 2015). Certain orders and suborders of arachnids have lost one type of eye altogether, with the homology of eyes clarified by the fossil record and embryology [31, 34, 35].

The second concern in extrapolating developmental processes derived from pancrustaceans is that a subset of Arachnida exhibits an ancient shared genome duplication, resulting in numerous paralogs of developmental patterning genes. Recent phylogenetic and comparative genomic works on Arachnida have shown that Arachnopulmonata [36–38], the clade of arachnids that bear book lungs (e.g., spiders, scorpions, whip spiders), retain duplicates of many key transcription factors, such as homeobox genes, often in conserved syntenic blocks [39–42]. Many of the ensuing paralogs exhibit non-overlapping expression patterns and a small number have been shown to have subdivided the ancestral gene function (subfunctionalization) or acquired new functions (neofunctionalization) [42–44].

While comparatively little is known about the genetics of arachnid eye development, gene expression surveys of insect retinal determination gene network (RDGN) homologs of two spiders (*Cupiennius salei* and *Parasteatoda tepidariorum*) have shown that this phenomenon extends to the formation of spider eyes as well [45, 46]. Different paralog pairs (orthologs of *Pax6*, *Six1*, *Six3*, *eyes absent*, *atonal*, *dachshund* and *orthodenticle*) exhibit non-overlapping expression boundaries in the developing eye fields, resulting in different combinations of transcription factor expression in the eye pairs [45, 46]. While these expression patterns offer a potentially elegant solution to the differentiation of spider eye pairs, only a few studies with the spider *P. tepidariorum* have attempted to experimentally test the role of these genes in the formation of arachnid eyes. *Ptep-orthodenticle-1* maternal RNA interference (RNAi) knockdown results in a range of anterior defects, including complete loss of the head, which precluded assessment of a role in the formation of the eyes [47]. *Ptep-dac2* RNAi knockdown results in appendage segment defects, but no eye patterning defects were reported by the authors [43]. More recently, a functional interrogation of both *Ptep-Six3* paralogs, focused on labrum development, reported no discernible morphological phenotype, despite a lower hatching rate than controls and disruption of a downstream target with a labral expression domain [48]. Thus, gene expression patterns of duplicated RDGN paralogs have never been linked to eye-related phenotypic outcomes in any arachnopulmonate model. Similarly, the functions of the single-copy orthologs of RDGN genes in groups like mites [49, 50], ticks [51], and harvestmen [35, 52, 53] are entirely unexplored, in one case because an otherwise tractable arachnid species lacks eyes altogether (the mite *Archegozetes longisetosus* [49, 54–56]).

Investigating the evolution of eye loss in arachnids thus has the potential to elucidate simultaneously (1) the morphogenesis of a poorly understood subset of metazoan eyes [31, 34], (2) developmental mechanisms underlying a convergent trait (i.e., eye loss in caves) in phylogenetically distant arthropod groups [5, 9], (3) shared programs in eye development common to Arthropoda (through comparisons with pancrustacean datasets) [26–29], and (4) the role of ancient gene duplicates in establishing the diversity of eyes in arachnopulmonates [42, 45, 46].

As first steps toward these goals, we developed transcriptomic resources for a sister species pair of cave-dwelling *Charinus* whip spiders, wherein one species exhibits typical eye morphology and the other highly reduced eyes (a troglobitic condition). We applied a differential gene expression (DGE) analysis to these datasets to investigate whether candidate RDGN genes with known expression patterns in model spider species (*C. salei*, *P. tepidariorum*) exhibit differential expression in non-spider arachnopulmonates, as a function of both eye condition and developmental stage. To link bioinformatic inference of expression patterns with functional outcomes, we interrogated the function of three candidate RDGN genes identified from DGE in a model arachnopulmonate, using RNAi in the spider *P. tepidariorum*, which exhibits the same number and types of eyes as whip spiders. We provide functional evidence that one of these candidates, *sine oculis/Six1*, is necessary for the development of all spider eye types.

## Results

### RDGN gene duplication in *Charinus* whip spiders

To investigate the possible role of Retinal Determination Gene Network (RDGN) genes in eye reduction in naturally occurring cave arachnids, we first generated transcriptomic resources for an empirical case of closely related, non-spider arachnopulmonate sister species pair that constitutes one epigean and one troglobitic species: the whip spider species *Charinus ioanniticus* Kritscher 1959 and *C. israelensis* (Fig. 1 B–C) [18]. Whip spiders, arachnopulmonates of the order Amblypygi, are commonly found in cave habitats ranging from rainforests, savannahs and deserts [57]. The recently described troglobitic species *C. israelensis* (reduced-eyes) occurs in close proximity to its congener *C. ioanniticus* (normal eyes) in caves in the Galilee, northern Israel [18]. Given that the formation of Levantine cave refuges is considerably recent, *C. israelensis* and *C. ioanniticus* are likely sister species with a short time of divergence, an inference supported by their similar morphology [18]. We collected ovigerous females from both species in caves in Israel and extracted RNA from embryos (Additional file 1, Table S1). Staging of the embryos and nomenclature of stages follows the description of whip spider embryology for the species *Phrynus marginemaculatus* [58, 59]. Our sampling focused on the deutembryo, the stage where most external features of the embryo, such as tagmosis and appendages are fully formed, but not the eyes. In *P. marginemaculatus*, the eyes begin to form around 50 dAEL, but the eye spots become externally visible and pigmented only close to hatching (90 dAEL) [58].

For de novo assembly of the embryonic transcriptomes of *C. ioanniticus* and *C. israelensis,* we extracted RNA from all deutembryo stages collected in the field (see Additional file 1, Table S1 for localities and sample explanations). Assemblies include two deutembryo stages before eyespot formation and one deutembryo stage bearing eyespots for *C. ioanniticus*; and two early deutembryo stages for *C. israelensis* (Additional file 1, Fig. S1). The assemblies of *C. ioanniticus* and *C. israelensis* exhibited an N50 of 1122 bp and 1045 bp, respectively (Additional file 1, Table S2); universal single copy ortholog benchmarking with BUSCO v3.0 [60] indicated completeness of 93.8 and 95.2%, respectively.

**Fig. 2:**
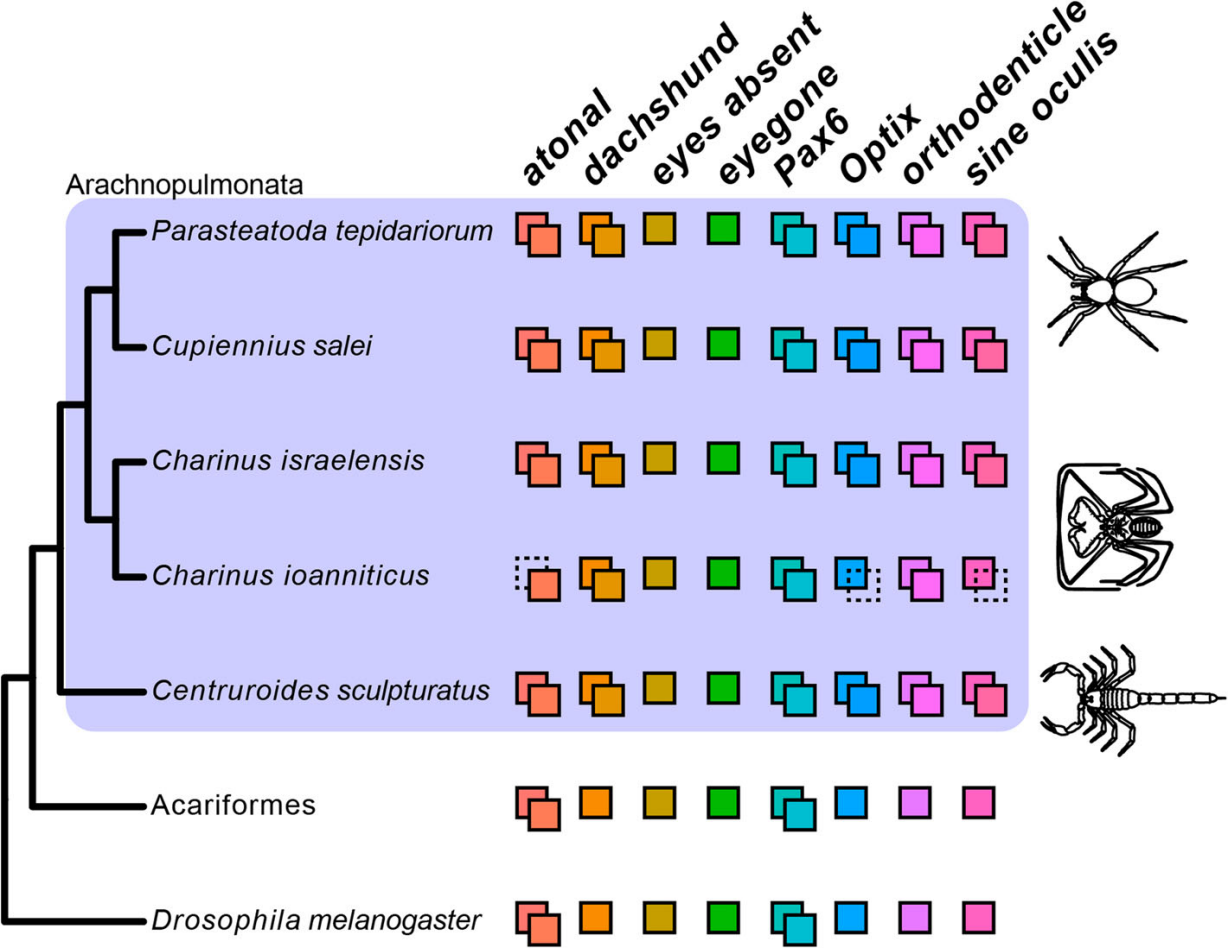
Phylogenetic distribution of Retinal Determination Gene Network (RDGN) homologs in an insect (*Drosophila melanogaster*), a non-arachnopulmonate arachnid group (Acariformes: *Dinothrombium tinctorium*; *Tetranychus urticae*) and Arachnopulmonata (spider: *Parasteatoda tepidariorum*; scorpion: *Centruroides sculpturatus*), including newly discovered orthologs in *Charinus* whip spiders (Amblypygi). Colored squares indicate number of paralogs for each RDGN gene. Dotted squares indicate presumed missing data, not gene loss. For comprehensive list of duplicated genes in Arachnopulmonata see Schwager et al. (2017) and Leite et al. 2018. Gene trees and alignments for each gene are available in Additional file 1 Dataset S1

Amblypygi is inferred to be nested stably in Arachnopulmonata, the clade of arachnids that bear book lungs [36, 37, 61–63]. Recent evidence suggests that the common ancestor of arachnopulmonates has undergone a whole- or partial-genome duplication affecting large gene families, such as homeobox genes [40–42]. The well-documented phylogenetic position of Amblypygi in Arachnopulmonata predicts that genes in RDGN that are duplicated in spiders, should also be duplicated in *Charinus* whip spiders (as well as other arachnopulmonate orders). To test this hypothesis, we performed phylogenetically-informed orthology searches on the newly assembled embryonic transcriptomes of both *Charinus* species, and conducted phylogenetic analysis with orthologs across selected arthropod species. We discovered that homologs of *atonal* (*ato*)*, Pax6*, *dachshund* (*dac*), *sine oculis* (*so*; *Six1*), *Optix* (*Six3*), and *orthodenticle* (*otd*) are duplicated in *Charinus*, whereas *eyegone* (*eyg*) and *eyes absent* (*eya*) occur as single-copy orthologs (these latter two also occurring single-copy in spiders) (Fig. 2). A detailed description of the orthology inference and annotation is available in the Additional file 1, Supplementary Results and Figures S2–S8. While the two copies of *ato* and *Pax6* are inferred to result from shared duplication with other arthropods (Fig. 2), the occurrence of paralogs of *dac*, *Optix*, *otd* and *so* in *Charinus* whip spiders, as well as a scorpion, suggests that the retention of RDGN ohnologs is systemic in Arachnopulmonata.

### RDGN gene expression differences related to eye formation in whip spiders: comparing early and late stages of *C. ioanniticus*

**Fig. 3:**
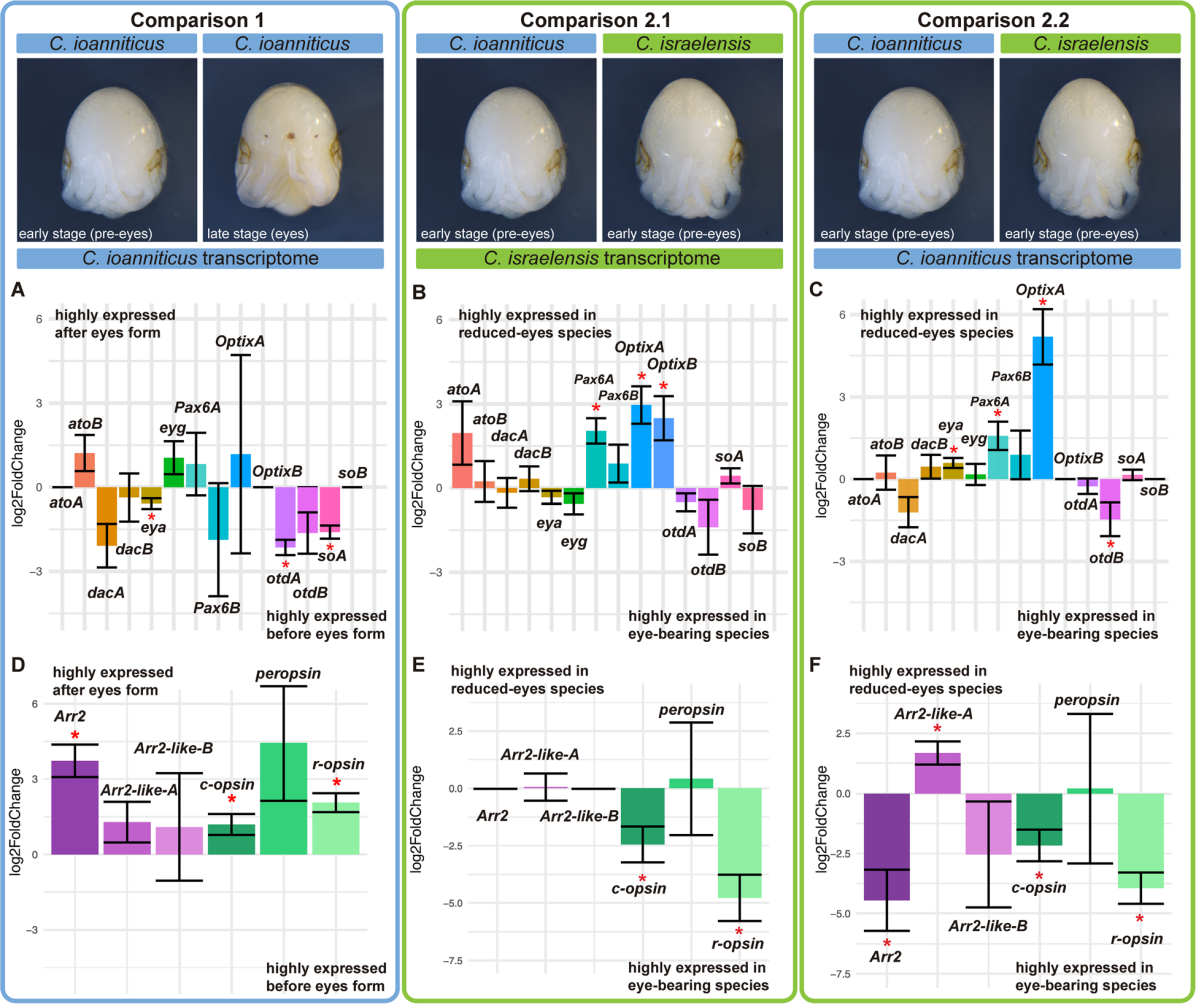
Differential gene expression analysis of Retinal Determination Gene Network (RDGN) genes and phototransduction genes in *Charinus* whip spider deutembryos. Bar graphs display log_2_ fold change of selected RDGN and phototransduction genes. The denominator of the differential gene expression is the always the sample in the left. **a**, **d**: Comparison 1; Comparison between reads of early (pre-eyespot) and late deutembryos (eyespot) of the eye-bearing species *C. ioanniticus* mapped onto *C. ioanniticus* transcriptome. **b**, **e**: Comparison 2.1; Comparison between reads of early deutembryo of *C. ioanniticus* and early deutembryo of *C. israelensis* mapped onto *C. israelensis* transcriptome. **c**, **f**: Comparison 2.2; Comparison between reads of early deutembryo of *C. ioanniticus* and early deutembryo of *C. israelensis* mapped onto *C. ioanniticus* transcriptome. *atoA*/*B*: *atonalA*/*atonalB*; *dacA*/*B*: *dachshundA*/*B*; *eya*: *eyes absent*; *eyg*: *eyegone*; *otdA*/*B*: *orthodenticleA*/*B*; *soA*/*B*: *sine oculisA*/*B*. *Arr2*: *Arrestin-2*; *Arr2-likeA/B*: *Arrestin-2-like A/B*. Asterisks denote genes that are differentially expressed with a p_adj_ > 0.05. Log_2_FC = 0 for *atoA*, *OptixB*, and *soB* for Comparison 1 and Comparison 2.2 are due to the absence of those paralogs in *C. ioanniticus* reference transcriptome. Log2FC = 0 for *Arr2* in Comparison 2.1 is due to absence of this gene in *C. israelensis* reference transcriptome

The expression of paralog pairs of *Pax6*, *so*, *Optix*, *eya*, *ato*, *dac*, and *otd* in the developing eyes of the spiders [45, 46], and the occurrence of the same paralogs in *Charinus* whip spiders, suggest that these genes may also be involved in the formation of eyes in whip spiders. We investigated this idea by comparing the expression levels of these RDGN genes in the stages before eye-spot formation versus a stage after eye-spot formation in the eye-bearing whip spider *C. ioanniticus* (henceforth “Comparison 1”; Fig. 3a, d).

We mapped reads of both treatments to the reference transcriptome of *C. ioanniticus* using the quasi-alignment software Salmon v. 1.1.0 [64] and conducted a differential gene expression analysis of Comparison 1 using DESeq2 v 1.24.0 [65] (Additional file 1, Fig. S9). These comparisons showed that *Cioa-otdA*, *Ciao-eya* and *Cioa-soA* are significantly more highly expressed (p_adj_ < 0.05) in the stage before eyespot formation in comparison with the stage with eyespots (Fig. 3a). The higher relative expression of both *so* and *eya* in that stage accords with the fact that in the fruit fly *D. melanogaster* they form a protein complex that regulates gene expression in synergy [66]. These relative expression dynamics in whip spiders are also consistent with the overlapping expression patterns of *eya* and *so* paralogs in the eyes of spiders [45, 46]. These results highlight the three RDGN genes as promising candidates involved in the formation of eyes in whip spiders.

### RDGN gene expression differences related to eye reduction in whip spiders: comparing *C. ioanniticus* and *C. israelensis*

Blindness in adults of the model cave fish *A. mexicanus* is a result of an embryonic process in which the rudimentary eye of the embryo is induced to degenerate by signals emitted from the lens tissue [67]. Both early and late expression of RDGN genes, such as *Pax6*, are responsible for the reduction of eyes in fish from cave populations [67, 68]. Likewise, in the isopod crustacean *A. aquaticus* cave blindness has a strong genetic component and mechanisms of eye reduction also act at embryonic stages [11, 69]. The embryonic development of the reduced-eyes whip spider *C. israelensis* has not been explored to date, but we expect that reduction of eyes results from changes in embryonic gene expression during the deutembryo stage [58]. We investigated this possibility by quantifying the relative gene expression of RDGN genes in comparable embryonic stages of *C. israelensis* (reduced eyes) and *C. ioanniticus* (normal eyes) embryos before eye-spot formation (Additional file 1 Table S1; Fig. S1). Using the DGE approach from Comparison 1, we conducted a heterospecific analysis using as the reference either the *C. israelensis* transcriptome (henceforth “Comparison 2.1”) or the *C. ioanniticus* transcriptome (henceforth “Comparison 2.2”).

Both analyses are anchored on the premise that a hybrid mapping between the sister species is possible given their recent divergence. The mapping rate of the *C. ioanniticus* reads was similar regardless of the reference species, (96.74 and 96.59% respectively for *C. ioanniticus* and *C. israelensis*). In the case of the reads from *C. israelensis* embryos, mapping rate to the conspecific (96.8%) transcriptome was higher than when mapping against *C. ioanniticus* (82.45%). The similar mapping rate of *C. ioanniticus* reads suggests that the two whip spiders are sufficiently closely related to generate interspecific comparisons of gene expression. Comparisons 2.1 and 2.2 yielded similar results with respect to the direction of differentially expressed RDGN genes (Fig. 3b–c). Intriguingly, Comparison 2.1 shows that *Pax6A*, *OptixA* and *OptixB* are significantly more highly expressed in the reduced-eyes species, with expression levels at least 4 times higher than in the normal-eyes species (log_2_FC > 2; p_adj_ < 0.05) (Fig. 3b; Additional file 1, Fig. S10). In Comparison 2.2, *Pax6A* and *OptixA* are also more highly expressed in *C. israelensis* (p_adj_ < 0.05), and so is *eya* (p_adj_ < 0.05; Fig. 3c). In Comparison 2.2, *otd-B* appears more highly expressed in the normal-eyes species (p_adj_ < 0.05; Fig. 3c; Additional file 1, Fig. S11). We note that the magnitude of log_2_FC and significance values differed considerably between analysis. Nonetheless, *Pax6A* and *OptixA* were consistently more highly expressed in the reduced-eyes species, highlighting these two genes as promising candidates involved in the reduction of eyes in *C. israelensis*.

#### Expression of phototransduction genes and gene ontology enrichment analysis

Some of the RDGN genes surveyed are pleiotropic and expressed outside the eye field in other arthropods, including arachnids. For instance, *dac* is an important appendage patterning gene [70] and *otd* regulates anterior patterning across Arthropoda [47, 71]. Therefore, our whole-embryo DGE comparisons may potentially not be sensitive enough to detect differences in expression in individual organs (i.e., eyes). In order to assess further the sensitivity of the approach to detecting eye-specific gene expression differences, we first quantified expression of opsins (visual pigments) and visual arrestins (phototransduction proteins) [72, 73]. We predicted that these retinal components should be up-regulated in the eye-spot stages of *C. ioanniticus*, and expected that embryos of this eyed species should have higher expression of retinal genes in comparison to *C. israelensis*, the reduced-eyes species. We found three transcripts annotated as opsins for *C. ioanniticus* and *C. israelensis*: a *r-opsin* (Long-wave-sensitive clade 2 [LWS-2]), a *peropsin* and a *c-opsin* (Additional file 1 Fig. S12). LWS-2 (also referred to as Rh2) and peropsins are expressed on the eyes of some chelicerates, while c-opsins have been reported only in the central nervous system [45, 74–77].

For the visual arrestins, we recovered one homolog of *D. melanogaster Arrestin-2* (*Arr2*) in *C. ioanniticus*. We did not recover orthologs of *Arrestin-1* (*Arr1*) in either *Charinus* transcriptome, but *Arr1* orthologs occur in the other chelicerate species surveyed (Additional file 1 Fig. S13). In addition, we discovered two *Arr2* paralogs in *C. ioanniticus* and *C. israelensis* that we termed *Arrestin-2-like* (A/B), given their close relationship to *Arr2* to the exclusion of *Arr1* and *D. melanogaster* non-visual arrestin *kutz* [78] (Additional file 1 Fig. S13).

In the intraspecific comparison between *C. ioanniticus stages* (Comparison 1), we detected that the *Arr2, c-opsin*, and *r-opsin* are significantly more highly expressed in the older stage with eye spots (Fig. 3d). In the interspecific Comparison 2.1, *c-opsin* and *r-opsin* are significantly more highly expressed in the eye bearing species (Fig. 3e). In Comparison 2.2, *Arr2, c-opsin*, and *r-opsin* are significantly more highly expressed in the eye bearing species (Fig. 3f). In this comparison, *Arrestin-2-like B* was more highly expressed in the reduced eye species, but we note that the identity of this arrestin needs to be further investigated, since it is does not cluster with the visual *Arr1* and *Arr2* (SI Appendix, Fig. S13). Taken together, these results suggest that our DGE approach is able to detect predicted differences in expression of downstream retinal genes between treatments.

Next, we conducted a Gene Ontology (GO) enrichment analysis in the gene sets of significantly highly and lowly expressed genes in the three comparisons, in order to investigate broader patterns of gene expression associated with eye development. We specifically looked for enrichment or depletion of the GO term “eye development” and child terms. In Comparison 1, we discovered enrichment of six eye-related GO terms only in the list of highly expressed genes in the stage before eyes form in *C. ioanniticus*, with 34 unique genes composing the enriched categories (Additional file 2, Table S3). These results accord with the detection of RDGN genes more highly expressed in the pre-eye stages (see above).

In Comparison 2.1, we detected ten eye-related enriched GO terms in the list of highly expressed genes in embryos of the blind species *C. israelensis*, with 187 unique genes composing the enriched categories (Additional file 2, Table S3). The enrichment analysis of Comparison 2.2 yielded congruent results (seven eye-related GO terms; 125 unique genes) (Additional file 2, Table S3). The detection of eye-related GO terms enriched only on the more highly expressed genes of *C. israelensis* was unexpected, but is in accordance with the relative higher expression of *Pax6A* and *OptixA* detected in the analysis of RDGN genes (see above).

#### sine oculis is necessary for principal and secondary eye development in a model arachnopulmonate.

Our bioinformatic analysis in the whip spider system suggested that *eya,* one paralog of *so*, and *otd* may be involved in the normal formation of eyes in *C. ioanniticus* (Comparison 1). We also found evidence that *Pax6* and a paralog of *Optix* may be involved in the reduction of eyes in the cave whip spider *C. israelensis*. To link bioinformatic measurements of gene expression with functional outcomes, we interrogated the function of RDGN genes using parental RNA interference (RNAi) in the spider *P. tepidariorum*. We selected *Ptep-soA* (*Ptep-so1* sensu Schomburg et al. 2015)*, Ptep-otdB* (*Ptep-otd2* sensu Schomburg et al. 2015) and *Ptep-OptixB* (*Ptep Six3.2* sensu Schomburg et al. 2015). In *P. tepidariorum*, these genes are known to be expressed in all eye types, in the median eyes only, and in the lateral eyes, respectively (Fig. 1d) [45].

**Fig. 4:**
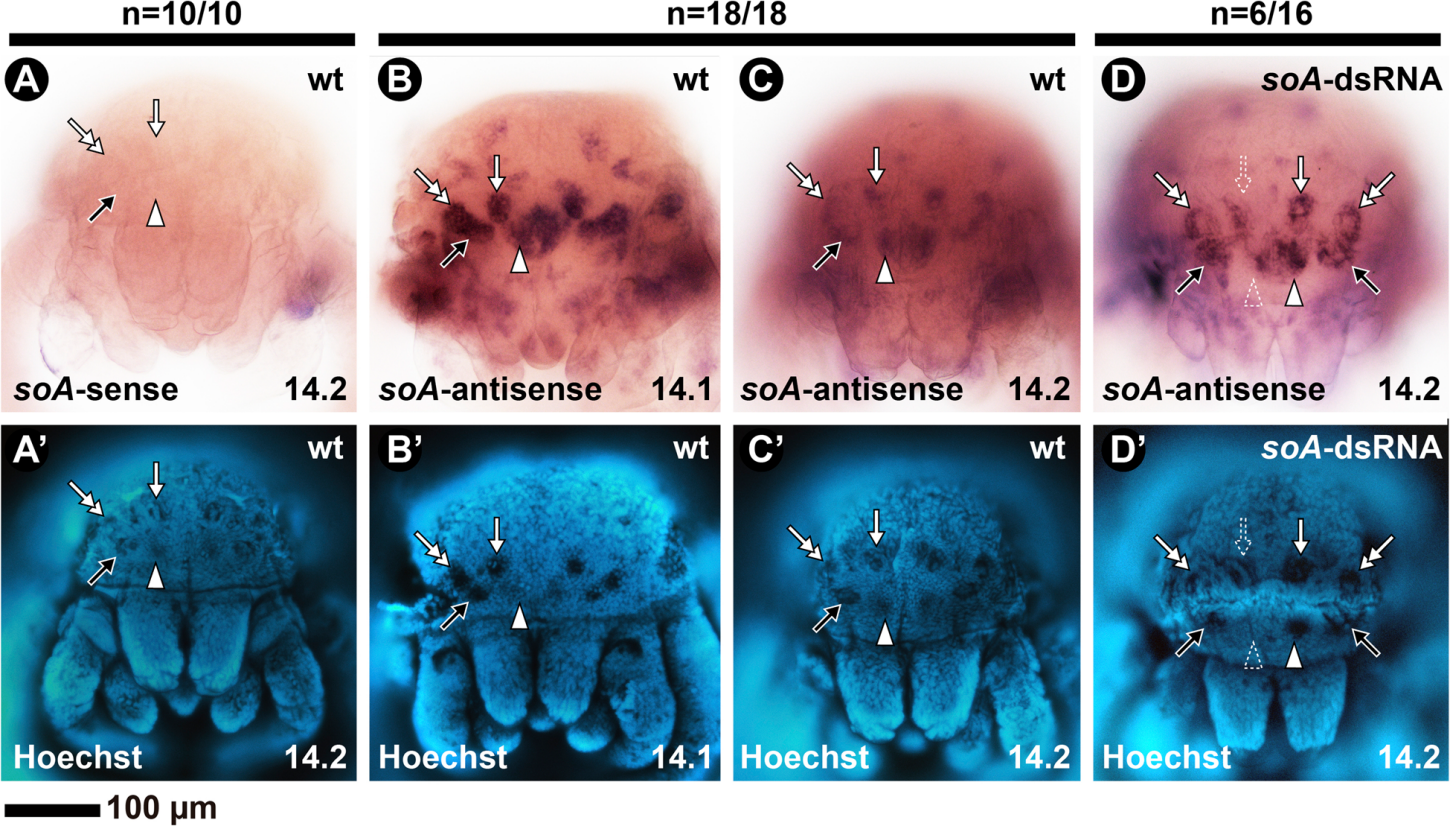
In situ hybridization using DIG-labeled riboprobes for *Ptep-soA* in late embryos of the spider *Parasteatoda tepidariorum*. All embryos in frontal view. **a**–**d**: bright field images. **A’**–**D’**: Same embryos, in Hoechst staining. **a**: Sense probe of a stage 14.2 embryo (no signal). **b**: Antisense probe on a wild type stage 14.1 embryo. **c**: Antisense probe on a wild type stage 14.2 embryo. **d**: Antisense probe on a stage 14.2 embryo from the *Ptep-soA* dsRNA-injected treatment. *soA*: *sine oculis A*. White arrowhead: median eye; Black arrow: anterior lateral eye; White arrow: median lateral eye; Double white arrow: Posterior lateral eye. Dotted arrowhead/arrow indicate asymmetrical expression and eye defect. Sample sizes are indicated above each treatment

Early expression of *Ptep-soA* is detected in lateral domains of the head lobes (stage 10) corresponding to the principal and secondary eyes, and continues until the pre-hatching stage 14 [45]. Expression of *Ptep-soA* on wild type stage 14.1 embryos is bilaterally symmetrical on all eyes and uniformly strong (Fig. 4a–b). By stage 14.2, it remains strong on the principal eyes but it is stronger at the periphery of the secondary eye spots (Fig. 4a, c).

*Parasteatoda tepidariorum* hatchlings, or postembryos, initially have no externally visible lenses and pigment. The red pigment and lenses of all eyes, and the reflective tapetum of the lateral eyes, become progressively recognizable in the 48 h (at 26 °C) until the animal molts into the first instar with fully formed eyes (Additional file 3, Video S1) (see also [79]). We fixed embryos from *Ptep-soA* dsRNA-injected and dH_2_O-injected treatments between 24 h–48 h, which encompasses stages where the eyes of postembryos are already recognizable until the first instar.

**Fig. 5:**
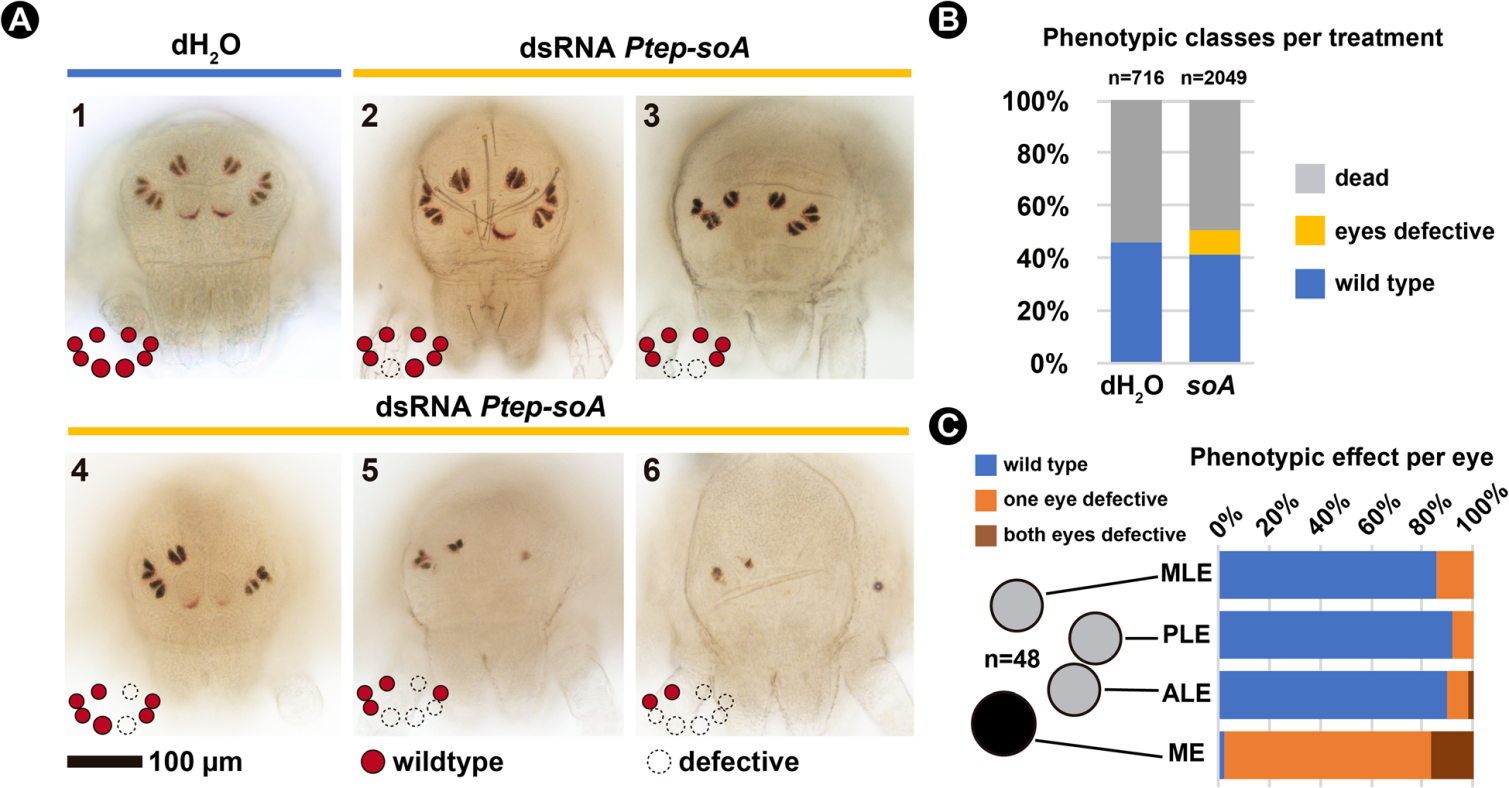
RNA interference against *Ptep-sine oculis A*. **a**: Bright field images of the spider *Parasteatoda tepidariorum* postembryos resulting from control treatment (dH_2_O-injected, panel 1) and double stranded RNA (dsRNA) injected treatment (panels 2–6), in frontal view. **b**: Frequencies of each phenotypic class per treatment from the combined clutches of all females. See Additional file 1 Fig. S15 for counts per clutch. **c**: Frequencies of symmetrical, asymmetrical, and wild type eyes quantified from a subset of 48 individuals with eye reduction phenotype. See Additional file 1 Fig. S14 for figures of all specimens and coding, and Methods for the scoring criteria. ME: median eyes; ALE: anterior lateral eyes; PLE: posterior lateral eyes; MLE: median lateral eyes. Schematics for the different eye types follows the nomenclature in Fig. 1

Negative control experiments (dH_2_O-injected females) yielded postembryos with eye morphology indistinguishable from wild type animals: the median eyes (ME; principal eyes) have an inferior semi-lunar ring of red pigment and lack the tapetum, and all pairs of lateral eyes (secondary eyes) have the canoe-shaped tapetum type [31, 32], which is split in the middle and surrounded by red pigment (Fig. 5a; panel 1). We observed misshaped tapeta on the lateral eyes of some postembryos on the earlier side of the developmental spectrum of fixed animals, but that was never observed on postembryos close to molting or first instars (Additional file 1, Fig. S14). It is unclear if this reflects a natural variation of early developing tapetum or an artifact of sample preparation.

Embryos from *Ptep-soA* dsRNA-injected females are also able to hatch into postembryos and continue molting to adulthood (Additional file 3, Video S2). However, a subset of the embryos of dsRNA-injected treatment (9.5%; *n* = 195/2049) exhibits a spectrum of eye defects that was not observed on the controls (Fig. 5a–b; Additional file 1, Fig. S15). The defects occurred on all eyes, namely median eyes (ME), anterior lateral eyes (ALE), posterior lateral eyes (PLE), and median lateral eyes (MLE) (Fig. 5a). Affected median eyes have reduced pigmentation or complete absence (Fig. 5a, panels 2–6), while lateral eyes also exhibited defects of the tapetum or complete absence of the eye (Fig. 5a, panels 4–6).

We selected a subset of the knockdown postembryos initially scored as having any eye defect (*n* = 48) for quantifying the degree of effect per eye type, and the proportion of symmetrical and mosaic eye phenotypes in our sample. Median eyes are affected in almost all cases (97%), whereas the three lateral eye types were similarly lowly affected (MLE: 14%; PLE: 8%; ALE: 10%) (Fig. 5c; Additional file 1, Fig. S14; detailed scoring criteria in Methods). The majority of defective eyes are mosaics, meaning that a given eye pair is affected only on one side of the animal (Fig. 5c; Additional file 1, Fig. S14).

Parental RNAi against *Ptep-soA* did not completely abolish its expression, as detected by in situ hybridization (Fig. 4d; see Methods). Nevertheless, we detected asymmetrical reduction of *Ptep-soA* expression on single eyes of a subset of stage 14 embryos (*n* = 6/16; Fig. 4d), which closely correlates with the predominance of mosaic phenotypes observed in late postembryos (Fig. 5c).

Parental RNAi experiments using the same protocol targeting *Ptep-otdB* and *Ptep-OptixB* did not result in any detectable phenotypic effects on the eyes of embryos from dsRNA-injected treatment (two and six females injected, respectively; counts not shown). These results accord with a recent study that knocked down both *Optix* paralogs *P. tepidariorum* and did not recover eye defects [48].

## Discussion

### Duplication of RDGN members in arachnopulmonates

Amblypygi have a critical placement within arachnid phylogeny, as they are part of a trio of arachnid orders (collectively, the Pedipalpi, comprised of Amblypygi, Thelyphonida, and Schizomida), which in turn is the sister group to spiders. Whereas the eyes of spiders have greatly diversified in structure, function, and degree of visual acuity (particularly the eyes of hunting and jumping spiders), the arrangement and number of eyes in Amblypygi likely reflects the ancestral condition across Tetrapulmonata (spiders + Pedipalpi), consisting of three pairs of simple lateral ocelli and a pair of median ocelli; a similar condition is observed in basally branching spider groups like Mesothelae and Mygalomorphae, as well as Thelyphonida (vinegaroons). However, while developmental genetic datasets and diverse genomic resources are available for spiders and scorpions [39, 41, 80, 81], the developmental biology of the other three arachnopulmonate orders has been virtually unexplored in the past four decades beyond the classic work describing the embryology of one North American amblypygid species [58] (but see two recent studies on developmental patterning genes in Amblypygi [59, 82]). To address this gap, we focused our investigation on a sister species pair of cave whip spiders and generated the first embryonic transcriptomes for this order. These datasets are immediately amenable to testing the incidence of RDGN duplicates previously known only from two spiders [45, 46] and their putative effects in patterning eyes across Arachnopulmonata broadly.

The inference of a partial or whole genome duplication (WGD) in the most recent common ancestor (MRCA) of Arachnopulmonata is supported by the systemic duplications of transcription factors and synteny detected in the genomes of the scorpion *Centruroides sculpturatus,* and the spider *P. tepidariorum*, as well as homeobox gene duplications detected in the genome of the scorpion *Mesobuthus martensii* and transcriptome of the spider *Pholcus phalangioides* [41, 42]. Additional evidence comes from shared expression patterns of leg gap gene paralogs in a spider and a scorpion [83]. Embryonic transcriptomes are particularly helpful in the absence of genomes, as several duplicated genes, such as some homeobox genes, are only expressed during early stages of development [39, 40, 42]. Our analysis of *Charinus* embryonic transcriptomes shows that RDGN gene duplicates observed in spiders also occur in whip spiders, supporting the hypothesis that these paralogs originated from a shared WGD event in the common ancestor of Arachnopulmonata. This inference is independently corroborated by the occurrence of arachnopulmonate-specific Hox gene and leg gap gene duplicates in both *Charinus* and *Phrynus* transcriptomes [59] as well as the occurrence of Wnt and frizzled gene duplicates [82].

The conservation of some transcription factors patterning eyes is widespread in the metazoan tree of life [84]. In the model fruit fly *D. melanogaster*, the homeobox Pax6 homolog *eyeless* was the first of several transcription factors identified as “master genes”, necessary for compound eye formation and capable of inducing ectopic eye formation [30, 85]. The Pax6 protein is essential for eye formation across several metazoan taxa, which has fomented ample debate about the deep homology of gene regulatory networks in patterning structurally disparate eyes [84, 86, 87]. In the case of *so* (Six1/2), orthologs are found across metazoans [88–90]. Evidence that *so* is required for the eye patterning in other bilaterians includes expression patterns in the developing eyes of the annelid *Platynereis dumerilii* [91], and functional experiments in the planarian *Girardia tigrina* [92]. Therefore, studies interrogating the genetic bases of eye formation in chelicerate models have the potential to clarify which components of the eye gene regulatory network of Arthropoda evolved in the MRCA of the phylum, and which reflect deep homologies with other metazoan genes.

### A conserved role for a *sine oculis* homolog in patterning arachnopulmonate eyes

The eyes of arthropods are diverse in number, arrangement, structure and function [93]. Both types of eyes observed in Arthropoda, the faceted eyes (compound) and single-lens eyes (ocelli), achieve complexity and visual acuity in various ways. To mention two extremes, in Mandibulata the compound eyes of mantis shrimps (Stomatopoda) achieve a unique type of color vision and movements by using 12 different photoreceptive types and flexible eye-stalks [94–96]. In Arachnida, the simple-lens median eyes of some jumping spiders (Salticidae) have exceptional visual acuity in relation to their eye size, achieve trichromatic vision through spectral filtering, and can move their retina using specialized muscles [32, 97, 98]. Comparative anatomy suggests that the common ancestor of Arthropoda had both lateral compound eyes and median ocelli that then became independently modified in the arthropod subphyla [34, 93]. In Chelicerata, the plesiomorphic eye condition is inferred to be a combination of median eyes (ocelli) and faceted eyes comparable to those of extant horseshoe crabs (Xiphosura), as well as extinct arachnid groups like Trigonotarbida and stem-group scorpions (e.g., *Proscorpius*) [93]. While in situ hybridization data for selected RDGN genes across arthropods generally support the hypotheses of eye homology, comparative developmental datasets remain phylogenetically sparse outside of Pancrustacea [45, 46].

We therefore applied a bioinformatic approach in a study system that lacked any genomic resources (Amblypygi) to assess whether RDGN homologs are transcriptionally active during the formation of eyes in the eye-bearing *C. ioanniticus*, as well as those that may be putatively involved in eye loss in its troglobitic sister species. As first steps toward understanding how arachnid eyes are patterned, our experiments demonstrated that *soA*, a *sine oculis* paralog identified as differentially expressed during the formation of eyes in *C. ioanniticus*, is necessary for patterning all eyes of a model arachnid system with the same eye configuration (*P. tepidariorum*). The reduction/loss of all eye types in the spider is consistent with the functional data in the beetle *T. castanaeum,* which demonstrates a role in compound eye formation (no ocelli occur in most beetles) [99], and in *D. melanogaster* and the cricket *G. bimaculatus*, in which both compound eyes and ocelli are affected [28, 30]. Thus, we provide the first functional evidence that part of the RDGN is evolutionarily conserved in the MRCA of insects and arachnids, and by extension, across Arthropoda.

The advantage of such a bioinformatic approach is that it can potentially narrow the range of candidate genes for functional screens, due to the inherent challenges imposed by duplications when assessing gene function. Eye reduction in the cave fish *A. mexicanus* has been shown to involve differential expression of genes known to be involved in eye patterning in model organisms, such as *hedgehog* and *Pax6* [5, 67]. In addition, other “non-traditional” candidates have been identified, such as *hsp90* [67]. Likewise, evidence from quantitative trait loci mapping in cave populations of the troglobitic crustacean *A. aquaticus* shows that eye loss phenotype is correlated with loci that are not part of the RDGN [5, 11]. The results of the DGE analysis in whip spiders underscore the potential of a DGE approach to triangulate targets among candidate genes in non-model species more broadly. Future efforts in the *Charinus* system should focus on dissecting individual eye and limb primordia of embryos of both species, in order to identify candidate genes putatively involved in the reduction of each eye type, as well as compensatory elongation of the sensory legs of the troglobitic species, toward downstream functional investigation.

### Do gene duplications play a role in the functional diversification of arachnopulmonate eyes?

A challenge in studying arachnopulmonate models to understand ancestral modes of eye patterning in Arthropoda is the occurrence of RDGN duplicates in this lineage. Our orthology searches and phylogenetic analysis showed that the evolutionary history of genes is not always resolved using standard phylogenetic methods, as short alignable regions and/or uncertainty of multiple sequence alignments can result in ambiguous gene trees. One way to circumvent this limitation is by analyzing expression patterns via in situ hybridization between paralogs in different arachnids in order to determine which patterns are plesiomorphic [42, 43, 83]. Nonetheless, the possibility of subfunctionalization and neofunctionalization may also complicate such inferences because discerning one process from the other is analytically challenging [100].

Genetic compensation of gene paralogs is another confounding variable; indeed, at least the redundancy of *Pax6* paralogs is inferred to be ancient in arthropods [101]. Deciphering the potentially overlapping or redundant functions of paralogs can be accounted for by experimental advances in model organisms (e.g., [102]), but comparable advances can be challenging for new systems. We note that the overall penetrance in our experiment is low (9.5%) when compared to some studies in *P. tepidariorum* (e.g., [103]; > 59% in *Ptep-Antp* RNAi). Wide variance in penetrance has been reported by several research groups in this system, with phenotypic effects varying broadly even within individual experiments (e.g., Fig. 5 of [104]; Fig. S5 of [105]). Furthermore, some genes have empirically proven intractable to transcript degradation by RNAi in *P. tepidariorum*, with one case suggesting functional redundancy to be the cause (posterior Hox genes [103];). Double knockdown experiments have been shown to exhibit poor penetrance (0–1.5%) in *P. tepidariorum* as well (Fig. S3 of [103]; Fig. S1 of [106]), and to our knowledge, no triple knockdown has ever been achieved. While we cannot rule out functional redundancy with other RDGN paralogs in the present study, the low penetrance we observed may also be partly attributable to our conservative phenotyping strategy (see Methods), which did not assess a possible delay in eye formation and emphasized dramatic defects in eye morphology for scoring.

The occurrence of RDGN gene duplications in Arachnopulmonata, in tandem with improving functional genetic toolkits in *P. tepidariorum* (e.g., [107]), offers a unique opportunity for studying the role of sub- and neofunctionalization during the development of their eyes, and a possible role for these processes in the diversification of number, position and structure of the eyes in an ancient group of arthropods [32, 34, 93, 97, 98]. Genomic resources for mites, ticks, and harvestmen [108–110] reveal that apulmonate arachnid orders have not undergone the genome duplication events exhibited by Arachnopulmonata [41] and separately by horseshoe crabs [111–113]. Future comparative studies focused on understanding the ancestral role of chelicerate RDGN genes should additionally prioritize single-copy orthologs in emerging model systems independent of the arachnopulmonate gene expansion, such as the harvestman *Phalangium opilio* [53, 114].

## Conclusions

Our work establishes a foundation to pursue the genetics of eye loss in cave arachnids, both by establishing a whip spider study system for comparative investigation, and by linking differential gene expression to an arthropod eye phenotype for the first time outside of Pancrustacea. Considering the phylogenetic position of arachnids, this finding implies that at least one of the classic eye genes discovered in insect model species had a conserved function in the common ancestor of Arthropoda. The systemic gene duplications in these arachnids offer a promising system for investigating the role of ohnologs in the diversification of arachnid eyes.

## Methods

### Animal collection

Three ovigerous females of the normal-eyes species, *C. ioanniticus* (ISR021–2; ISR021–3; ISR021–4), and two egg-carrying females of the reduced-eyes species, *C. israelensis* (ISR051–4; ISR051–6), were hand collected in caves in Israel in August 2018 (Supplementary Information; Table 1). Females were sacrificed and the brood sacs containing the embryos were dissected under phosphate saline buffer (PBS). For each female, a subset of the embryos (5 to 13 individuals) was fixed in RNAlater (ThermoFisher) after poking a whole into the egg membrane with fine forceps, while the remaining embryos of the clutch were fixed in a 4% formaldehyde/PBS solution to serve as vouchers (Additional file 1, Table S1). Adult animals and embryos of *P. tepidariorum* were obtained from the colony at UW-Madison, US, in turn derived from a laboratory culture founded with spiders collected near Cologne, Germany [115].

### Transcriptome assembly for *Charinus* whip spiders

RNA*later*-fixed embryos were transferred to 1.5 mL tubes filled with TRIZOL (Invitrogen) after 2 months, and subject to RNA extraction. Total RNA extracted from each sample of the embryos of *C. ioanniticus* (three samples) and *C. israelensis* (two samples) (Additional file 1, Table S1) was submitted for library preparation at the Biotechnology Center of the University of Wisconsin-Madison. Each sample was sequenced in triplicate in an Illumina High-Seq platform using paired-end 100 bp-long read strategy at the same facility. Read quality was assessed with FastQC (Babraham Bioinformatics). Paired-end reads for *C. ioanniticus* (ISR021) and *C. israelensis* (ISR051) were compiled and de novo assembled using Trinity v.3.3 [116] enabling Trimmomatic v.0.36 to remove adapters and low-quality reads [117]. Transcriptome quality was assessed with the Trinity package script ‘*TrinityStats.pl*’ and BUSCO v.3 [60]. For BUSCO, we used the ‘Arthropoda’ database and analyzed the transcriptomes filtered for the longest isoform per Trinity gene.

### RNA sequencing for differential gene expression

The total RNA extraction of each sample of *C. ioanniticus* and *C. israelensis* embryos was sequenced in triplicate in an Illumina High-Seq platform using a single-end 100 bp-long read strategy in the same facility as described above. For *C. ioanniticus* (normal-eyes), we sequenced two biological replicates of embryos at an early embryonic stage, before eye-spot formation (ISR021–2, ISR021–3), and one sample of late embryos, after eye-spot formation (ISR021–4); For *C. israelensis* (reduced-eyes), we sequenced embryos at an early embryonic stage (ISR051–6; ISR051–4) comparable to the early stage in *C. ioanniticus* (ISR021–2, ISR021–3), as inferred by the elongated lateral profile of the body and marked furrows on the opisthosomal segments (Additional file 1, Fig. S1).

### Differential gene expression analysis in *Charinus* and identification of eye gene orthologs

Orthologs of *D. melanogaster* *ey* and *twin of eyeless* (*Pax6A, Pax6B*), *sine oculis* (*soA*, *soB*), *orthodenticle* (*otdA*, *otdB*), *Optix* (*Six3.1*, *Six3.2*), *dachshund* (*dacA*, *dacB*), and *eyes absent* (*eya*) had been previously isolated in *P. tepidariorum* (Schomburg et al., 2015, and references therein). We used as reference sequences the complete predicted transcripts for these genes from *P. tepidariorum* genome [41], *Cupiennius salei* [46] (for *atonal* [ato] and *Pax6*), and *D. melanogaster*, including also *ato* and *eyegone* (*eyg*) from the latter species. The sequences were aligned with MAFFT (v7.407) [118] and the resulting alignments were used to build hidden Markov model profiles for each gene (hmmbuild, from the hmmer suite v.3.3) [119]. Matches to these profiles were found using hmmsearch in the reference transcriptomes of *C. ioanniticus* and *C. israelensis* as well as in the genomes of representative arthropods, including *D. melanogaster* (GCA 000001215.4), *T. castaneum* (GCA 000002335.3), *Daphnia magna* (GCA 003990815.1), *Strigamia maritima* (GCA 000239455.1), *Dinothrombium tinctorium* (GCA 003675995.1), *Ixodes scapularis* (GCA 002892825.2), *Tetranychus urticae* (GCA 000239435.1), *Limulus polyphemus* (GCA 000517525.1), *Tachypleus tridentatus* (GCA 004210375.1), *C. sculpturatus* (GCA 000671375.2), *P. tepidariorum* (GCA 000365465.2) and *Trichonephila clavipes* (GCA 002102615.1). These species were selected from a pool relatively recent genome assembly resources and well curated reference genomes.

Homologous sequences (those with hmmer expectation value, e < 10^10^) to the genes of interest were then compiled into individual gene FASTA files, combined with the reference sequences used for the homology search, aligned (MAFFT [118]), trimmed of gap rich regions (trimAL v.1.2, −gappyout) [120] and used for maximum likelihood gene tree estimation (IQTREE v.1.6.8, −mset LG,WAG,JTT,DCMUT –bb 1000) [121]. The association of transcripts in the *Charinus* species with the genes of interest is based on the gene phylogeny and was followed by inspection of the coding sequences to distinguish splicing variants from other gene paralogs. Alignments, newick trees, and the list of *Charinus* sequences are available in Additional file 4 Dataset S1. The gene transcript association was then used to generate the transcript-to-gene map required for the DGE analysis.

For the analysis of opsins, protein sequences of the five *P. tepidariorum* opsins identified in a previous study [45] were used as queries for tblastn searches, and candidates were reciprocally blasted against NCBI non-redundant sequence database. For identification of arrestin homologs, the same procedure was performed using *D. melanogaster Arr1 *(FBpp0080583) and *Arr2* (FBpp0076326) as queries. Protein sequences of metazoan opsins from previous studies [34, 75] were aligned with candidate opsins (MAFFT [118]), and gene trees were inferred in a maximum likelihood phylogenetic analysis (IQTREE v.1.6.8, −m TEST –bb 1000). The annotation of arrestins was based on the nomenclature and reference protein sequences in [72], supplemented with arrestins identified by blastp in the genomes of *T. castaneum*, *L. polyphemus*, *P. tepidariorum*, *C. sculpturatus*, *I. scapularis* and *T. urticae*. A *Homo sapiens* alfa arrestin (NP_056498.1) was used as a reference outgroup. Alignment and gene tree inference were performed as above.

### Read mapping, transcript abundance quantification, and GO enrichment analysis

For the *in silico* analysis of gene expression, single-end raw reads were first trimmed using the software Trimmomatic v. 0.35 [117]. For the intraspecific analysis of early (before eyespot) and late (eyespot) embryos of *C. ioanniticus* (Comparison 1), the trimmed reads were quantified in the embryonic transcriptome of *C. ioanniticus*. For the interspecific comparison of early embryos of *C. ioanniticus* and *C. israelensis*, two reciprocal analysis were conducted: reads from both species mapped onto *C. israelensis* transcriptome as the reference (Comparison 2.1); and reads from both species mapped onto *C. ioanniticus* transcriptome (Comparison 2.2).

Transcript abundance was quantified using the software Salmon v. 1.1.0 [64], enabling the flag ‘–*validateMapping*’. Analysis of differential gene expression was conducted with the software DESeq2 v 1.24.0 [65] following a pipeline with the R package *tximport* v.1.12.3 [122]. The exact procedures are documented in the custom R script (Additional file 5, Dataset S2).

For the enrichment analysis, we annotated both transcriptomes using the Trinotate v.3.2.1 pipeline [123] and extracted GO term annotations with ancestral GO terms using the package script *extract_GO_assignments _from_Trinotate_xls.pl*. We conducted the GO enrichment analysis using the R package Goseq v.1.40.0 [124], as implemented by a modified Trinity v.2.8.5 script *run_GOseq.pl* [125]. Enrichment analyses were conducted for Comparison 1, Comparison 2.1 and Comparison 2.2, separately for the up-regulated (log_2_FC > 1) and down-regulated (log_2_FC < 1) set of significant genes (p_adj_ ≤ 0.05). We considered a GO term enrichment or depleted if FDR ≤ 0.05 (Additional file 6, Dataset S3). We searched for enriched GO terms associated with eye development (GO:0001654), and daughter GO terms (177 GO identifiers) as retrieved by the function get_child_nodes in R package GOfuncR v.1.8.0 [126].

### Parental RNA interference, in situ hybridization, and imaging in *Parasteatoda tepidariorum*

Total RNA from a range of embryonic stages of *P. tepidariorum* was extracted with TRIZOL (Invitrogen), and cDNA was synthetized using SuperScriptIII (Invitrogen). Gene fragments for *Ptep-soA*, *Ptep-otdB*, and *Ptep-OptixB* were amplified from cDNA using gene specific primers designed with Primers3Web version 4.1.0 [127] and appended with T7 ends. Cloning amplicons were generated using the TOPO TA Cloning Kit with One Shot Top10 chemically competent *Escherichia coli* (Invitrogen). Amplicon identities and directionality were assessed with Sanger sequencing. Primer, amplicon sequences and fragment lengths are available in Additional file 7 Dataset S4. Double-stranded RNA for *Ptep-soA*, *Ptep-otdB* and *Ptep-OptixB* was synthesized using the MEGAScript T7 transcription kit (Thermo Fisher). Sense and antisense RNA probes for colorimetric in situ hybridization were synthesized from plasmid templates with DIG RNA labeling mix (Roche) and T7/T3 RNA polymerase (New England Biolabs).

Parental RNA interference (RNAi) followed established protocols for double-stranded RNA (dsRNA) injection in virgin females of *P. tepidariorum* [81]. Each female was injected four times with 2.5 μL of dsRNA at a concentration of 2 μg/uL, to a total of 20 μg. For *Ptep-soA*, seven virgin females were injected with dsRNA of a 1048 bp cloned fragment (Additional file 1, Fig. S15C) and 3 females were injected with the same volume of dH_2_O as a procedural control. Two virgin females were injected with dsRNA for *Ptep-otdB*, and six females for *Ptep-OptixB*. All females were mated after the second injection and were fed approximately every-other day after the last injection. Cocoons were collected until the sixth clutch, approximately once per week.

Hatchlings for all cocoons were fixed between 24 and 48 h after hatching. Freshly hatched postembryos have almost no external signs of eye lenses and pigments. The selected fixation window encompasses a period in which postembryos have deposited eye pigments until the beginning of the first instar, where eyes are completely formed (Additional file 3, Video S1, S2). Hatchlings were immersed in 25% ethanol/PBST and stored at 4 °C. For the *Ptep-soA* RNAi experiment, hatchlings were scored in four classes: (1) wild type, where all eyes were present and bilaterally symmetrical; (2) eyes defective, where one or more eyes were reduced in size or completely absent; (3) dead/arrested; (4) undetermined, where embryos were damaged or clearly freshly hatched. A subset of *Ptep-soA* dsRNA-injected embryos from four clutches (*n* = 48) and of three control clutches (*n* = 48) were further inspected in detail to assess the effects on individual eye types. Given that there is a spectrum on the intensity of pigment deposition in the median eyes (ME), and small asymmetries on the shape of the early developing tapetum of the lateral eyes (LE) in control embryos, the following conservative criteria were adopted: (1) ME were considered affected when asymmetry in pigmentation or lens size was detected; both ME were only scored as affected when they were both completely missing, in order to rule out embryos that were simply delayed in pigment deposition; (2) LE were considered defective only when the tapetum was completely absent (Additional file 1, Fig. S14). Therefore, our coding does not allow detection of a phenotype consisting of delayed pigmentation. Raw data are available in Additional file 7 Dataset S4.

For in situ hybridization, a subset of *Ptep-soA* dsRNA-injected embryos at stage 13/14 [79] was fixed in a phase of heptane and 4% formaldehyde for 12–24 h, washed in PBST, gradually dehydrated in methanol and stored at − 20 °C for at least 3 days before downstream procedures, after a modified protocol of Akiyama-Oda and Oda (2003). In situ hybridization followed the protocol of Akiyama-Oda and Oda (2003).

Embryos from in situ hybridization were counterstained with Hoechst 33342 and imaged using a Nikon SMZ25 fluorescence stereomicroscope mounted with a DS- Fi2 digital color camera (Nikon Elements software). For postembryos, the prosoma was dissected with fine forceps, gradually immersed in 70% Glycerol/PBS-T and mounted on glass slides. Postembryos were imaged using an Olympus DP70 color camera mounted on an Olympus BX60 epifluorescence compound microscope.

The datasets in Additional files 3, 4, 5, 6 and 7 are deposited in: https://datadryad.org/stash/share/xb2VW4o80AmId3mLFZB07Ho7rHxPx-htK9q5J_-2miM

doi:10.5061/dryad.xgxd254d1

## Supplementary Information

**Additional file 1.** Figs. S1–S15 and Tables S1–S2. (.pdf)

**Additional file 2.** Table S3: Subset of enriched GO terms that have eye-related ontology for Comparison 1, Comparison 2.1, and Comparison 2.2. Each spreadsheet is accompanied by the Trinotate annotation report of the differentially expressed genes in the enriched eye-related GO terms. GO enrichment analyses absent from this file do not have eye-related GO terms enriched. For the full GOseq results for each comparison see Additional file 6, Dataset S3. (.xlsx)

**Additional file 3: **Video S1: Time-lapse imaging of a postembryo ~ 24 h after hatching of *Parasteatoda tepidariorum* from the dH_2_O-injected treatment (negative control). Pictures were taken every 30 min, in a room at 22 °C. Normal molting time after hatching is ~ 48 h at 26 °C. Video S2: Time-lapse imaging of a postembryo ~ 24 h after hatching of *Parasteatoda tepidariorum* from the *Ptep-soA*-injected treatment. Pictures were taken every 30 min, in a room at 22 °C. Normal molting time after hatching is ~ 48 h at 26 °C. (.zip). Available at: https://datadryad.org/stash/share/xb2VW4o80AmId3mLFZB07Ho7rHxPx-htK9q5J_-2miM; doi:https://doi.org/10.5061/dryad.xgxd254d1

**Additional file 4.** Dataset S1: Dataset of the orthology analyses. Alignments and sequences of *Charinus* RDGN genes identified in this study and gene trees in Newick format; alignments of opsins and arrestins. (.zip). Available at: https://datadryad.org/stash/share/xb2VW4o80AmId3mLFZB07Ho7rHxPx-htK9q5J_-2miM; doi:https://doi.org/10.5061/dryad.xgxd254d1

**Additional file 5.** Dataset S2: Dataset for the differential gene expression analyses. DESeq2 dataset (*DESeq (dds)*; filtered for p_adj_ > 0.5) of the DGE analysis of Comparison 1, 2.1 and 2.2 (see “Material and Methods” for explanation). *run_ddseq2.r*: custom R script used to run all three analysis. (.zip). Available at: https://datadryad.org/stash/share/xb2VW4o80AmId3mLFZB07Ho7rHxPx-htK9q5J_-2miM; doi:https://doi.org/10.5061/dryad.xgxd254d1

**Additional file 6.** Dataset S3: Dataset for the GOseq enrichment analyses. This folder contains the complete GOseq results of enriched/depleted (FDR ≤ 0.05) GO terms of up- (Log_2_FC > 1) and down-regulated (Log_2_FC < 1) genes in Comparisons 1, 2.1 and 2.2. The twelve spreadsheets (.xls) are named as follows: Comparison#,_UP/DOWN_enriched/depleted. (.zip). Available at: https://datadryad.org/stash/share/xb2VW4o80AmId3mLFZB07Ho7rHxPx-htK9q5J_-2miM; doi:https://doi.org/10.5061/dryad.xgxd254d1

**Additional file 7.** Dataset S4. (a): High resolution Additional file 1 Fig. S14. (b): Spreadsheets with the raw counts and sum of counts used to generate the distribution bar plots of the *Ptep-soA* RNAi experiment. (c) raw counts and sum of counts used to generate the distribution bar plots of the effects of *Ptep-soA* RNAi per eye type. (d) Primer sequences for the amplified fragments of *Ptep-soA*, *Ptep-otdB* and *Ptep-OptixB*. (.zip). Available at: https://datadryad.org/stash/share/xb2VW4o80AmId3mLFZB07Ho7rHxPx-htK9q5J_-2miM; doi:https://doi.org/10.5061/dryad.xgxd254d1

## Data Availability

The datasets generated and/or analyzed during the current study are available in the Additional files 2–7 Videos and Datasets in the Dryad repository (https://datadryad.org/stash/share/xb2VW4o80AmId3mLFZB07Ho7rHxPx-htK9q5J_-2miM). Raw reads are deposited in NCBI SRA database under accession number PRJNA649577.

## Abbreviations

*atoA*/*B*: *atonalA*/*atonalB*
*dacA*/*B*: *dachshundA*/*B*
*eya*: *eyes absent*
*eyg*: *eyegone*
*otdA*/*B*: *orthodenticleA*/*B*
RDGN: Retinal Determination Gene Network
*soA*/*B*: *sine oculisA*/*B*
ME: Median eyes
ALE: Anterior lateral eyes
PLE: Posterior lateral eyes
MLE: Median lateral eyes
LE: Lateral eyes
