# Supplementary material for: Systemic paralogy and function of retinal determination network homologs in arachnids
Source: BMC Genomics. 2020 Nov 23;21:811. doi: 10.1186/s12864-020-07149-x (PMC7681978; doi:10.1186/s12864-020-07149-x)
Supplement: Supplementary file 1 — Additional file 1. Figs. S1–S15 and Tables S1–S2. (.pdf) [file 12864_2020_7149_MOESM1_ESM.docx]

**Systemic paralogy and function of retinal determination network homologs in arachnids**

Authors:

*Guilherme Gainett, *Jesús A. Ballesteros, Charlotte R. Kanzler, Jakob T. Zehms, John M. Zern, Shlomi Aharon, Efrat Gavish-Regev, Prashant P. Sharma

*Equal author contribution

Correspondence: ggainett@gmail.com, ballesterosc@wisc.edu

Table of Contents

[Additional file 1, Results 2](#_Toc51867282)

[Additional file 1, Figure S1 5](file:////Users/gainett/Google_Drive/PhD/3_Cave%20blindness/Submisson_BMC_Genomics/Resubmission/2nd_review/Additional_file_1.docx#_Toc51867283)

[Additional file 1, Figure S2: 6](file:////Users/gainett/Google_Drive/PhD/3_Cave%20blindness/Submisson_BMC_Genomics/Resubmission/2nd_review/Additional_file_1.docx#_Toc51867284)

[Additional file 1, Figure S3: 7](file:////Users/gainett/Google_Drive/PhD/3_Cave%20blindness/Submisson_BMC_Genomics/Resubmission/2nd_review/Additional_file_1.docx#_Toc51867285)

[Additional file 1, Figure S4: 8](file:////Users/gainett/Google_Drive/PhD/3_Cave%20blindness/Submisson_BMC_Genomics/Resubmission/2nd_review/Additional_file_1.docx#_Toc51867286)

[Additional file 1, Figure S5: 9](file:////Users/gainett/Google_Drive/PhD/3_Cave%20blindness/Submisson_BMC_Genomics/Resubmission/2nd_review/Additional_file_1.docx#_Toc51867287)

[Additional file 1, Figure S6: 10](file:////Users/gainett/Google_Drive/PhD/3_Cave%20blindness/Submisson_BMC_Genomics/Resubmission/2nd_review/Additional_file_1.docx#_Toc51867288)

[Additional file 1, Figure S7: 11](file:////Users/gainett/Google_Drive/PhD/3_Cave%20blindness/Submisson_BMC_Genomics/Resubmission/2nd_review/Additional_file_1.docx#_Toc51867289)

[Additional file 1, Figure S8: 12](file:////Users/gainett/Google_Drive/PhD/3_Cave%20blindness/Submisson_BMC_Genomics/Resubmission/2nd_review/Additional_file_1.docx#_Toc51867290)

[Additional file 1, Figure S9: 13](file:////Users/gainett/Google_Drive/PhD/3_Cave%20blindness/Submisson_BMC_Genomics/Resubmission/2nd_review/Additional_file_1.docx#_Toc51867291)

[Additional file 1, Figure S10: 14](#_Toc51867292)

[Additional file 1, Figure S11: 15](#_Toc51867293)

[Additional file 1, Figure S12: 17](#_Toc51867294)

[Additional file 1, Figure S13: 18](#_Toc51867295)

[Additional file 1, Figure S14: 20](#_Toc51867296)

[Additional file 1, Figure S15: 21](#_Toc51867297)

[Additional file 1, Table S1: 23](#_Toc51867298)

[Additional file 1, Table S2: 24](#_Toc51867299)

# Additional file 1, Results

*Gene identification: RDGN genes*

*atonal*: The *atonal* gene tree showed poor resolution (Additional file 1, Fig. S2), hampering unambiguous assignment of the whip spider genes to *atonal* copies previously annotated in spiders [1,2]. *D. melanogaster* copies of *atonal* and *amos* clustered together forming a clade with other pancrustacean and myriapod sequences, suggesting these paralogs are restricted to Mandibulata. The fruit fly *cousin of atonal* (*cato*) formed a clade including the *Cupiennius salei* sequence of *atonalB* whereas the second copy of *C. salei*, *atonalA*, is found in an independent clade with only arachnid sequences. It is in this later clade that the only sequences of *Charinus* related to *atona*l are found, in turn forming two separate clades with clear amino acid differences between these copies (Additional file 4, Dataset S1; *atonal* alignment). Herein, these copies are labeled *atonalA* (*atoA*) and *atonalB* (*atoB*). Note that the reference genomic sequences, annotated as “*atonal like homolog 8 like*" (Ptep XP 0159181091), is found orthologous to the gene *net* in *D. melanogaster*.

*Pax6*: In *D. melanogaster*, there are two paralogous copies of the vertebrate *Pax6*, *eyeless* and *twin of eyeless*, and this duplication seems to be shared across Arthropoda [3]. Both *Pax6* copies have been characterized in spiders [1,4], and two copies occur in all arachnids investigated here except the in mite *Tetranychus urticae* (Additional file 1, Fig. S3). The gene tree of *Pax6* homologues clearly identified a clade for *toy* including chelicerate and mandibulate copies, but no *Charinus* sequences are found in this clade (Additional file 1, Fig. S3). The sister clade (*eyeless*) consists only of pancrustacean sequences whereas the chelicerate copies, previously annotated as *eyeless* orthologs, are found in a separate clade. Among these, two distinct genes, herein dubbed *Pax6A* and *Pax6B*, are present in both *Charinus* species. Sequence similarity searches (blastp) of both *Pax6A* and *Pax6B* against the genome of *Drosophila melanogaster* points to *Dmel-toy* as the best hit, followed by *Dmel-ey*. Therefore, although the homology of these copies with *Dmel-ey/toy* is evident, it is not trivial to assign these to either of these genes or if these represent taxon-restricted duplicates of *eyeless*.

*eyegone/twin of eyegone*: *Pax6* is represented by two homologs in *D. melanogaster* (*eyegone/twin of eyegone* ) versus a single copy in arachnids. Single copy orthologs of eyg/toe are present in the two target Amblypygi species (Additional file 1, Fig. S3).

*dachshund*: Spiders and scorpions have two paralogous copies of *dachshund* [5,6]. Two copies are present in the transcriptomes of both *Charinus* species and are here termed *dacA* and *dacB* (Additional file 1, Fig. S4). The *C. israelensis dacB* is assembled in two different gene fragments that overlap by three amino acids (Additional file 1, Fig. S4; see *dachshund* alignment in Additional file 4, Dataset S1). The *C. ioanniticus* *dacA* copy is also assembled as two different gene fragments with little sequence overlap but being part of the *dacA* clade (Additional file 1, Fig. S4).

*eyes absent*: This single-copy ortholog is found in arthropods and arachnids alike and is represented in both *Charinus* species. The annotation of transcripts to this gene is unambiguous for both amblypigid species (Additional file 1, Fig. S5).

*orthodenticle*: As with spiders, there are two copies homologous to *Dmel-otd* in *Charinus*. Two copies are also present in other non-dipteran mandibulates [7] (Additional file 1, Fig. S6). The resolution of the gene tree is poor and does not allow uncontroversial association to spider orthologs (Additional file 1, Fig. S6). *Charinus* copies are termed *otdA* and *otdB*.

*Optix*: There are two very similar copies of *Optix* in *C. israelensis* and one in *C. ioanniticus* (Additional file 1, Fig. S7). The *C. ioanniticus* copy is termed *OptixA*. The two copies of *C. israelensis* show very conserved amino acid sequences but clear nucleotide differences. Although the gene tree with the reference genome shows them more closely allied to one of the spider paralogous copies of *Optix* (Ptep NP 00130752.1), a reduced analysis including *Cupiennius salei* and *P. tepidariorum* copies, suggests that the *Charinus* copies are independent duplications. Here the two whip spider copies are dubbed *OptixA* and *OptixB* but they should not be considered orthologous to the spider *OptixA/B*.

*sine oculis:* Two copies of *sine oculis* are found in *C. israelensis* and one in *C. ioanniticus*. Both copies are nested in a clade with *Ptep-soA* (Additional file 1, Fig. S8). These are herein dubbed as *soA* and *soB* given that orthology with either spider copy is unclear.

**
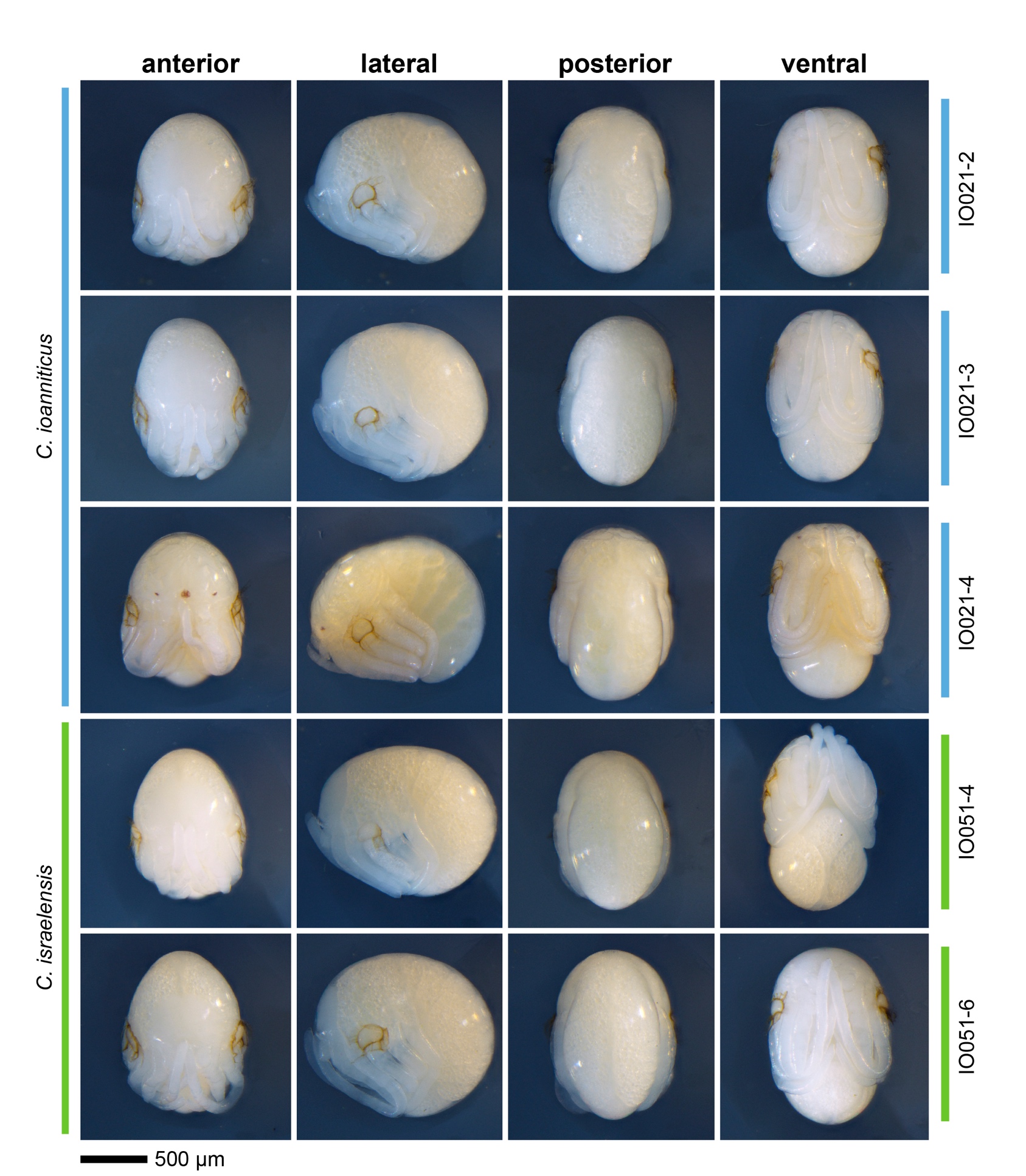
**

Additional file 1, Figure S1**:** Voucher specimens of the deutembryo stages Charinus ioanniticus (top three rows) and Charinus israelensis (top bottom rows) used in each RNA extraction. Each row is a different view of the same embryo. IO051-2 and IO051-3: early deutembryos pre-eyespots, C. ioanniticus. IO021-4: late deutembryos with eyespots, C. ioanniticus. IO051-4 and IO051-6: early deutembryos.

**
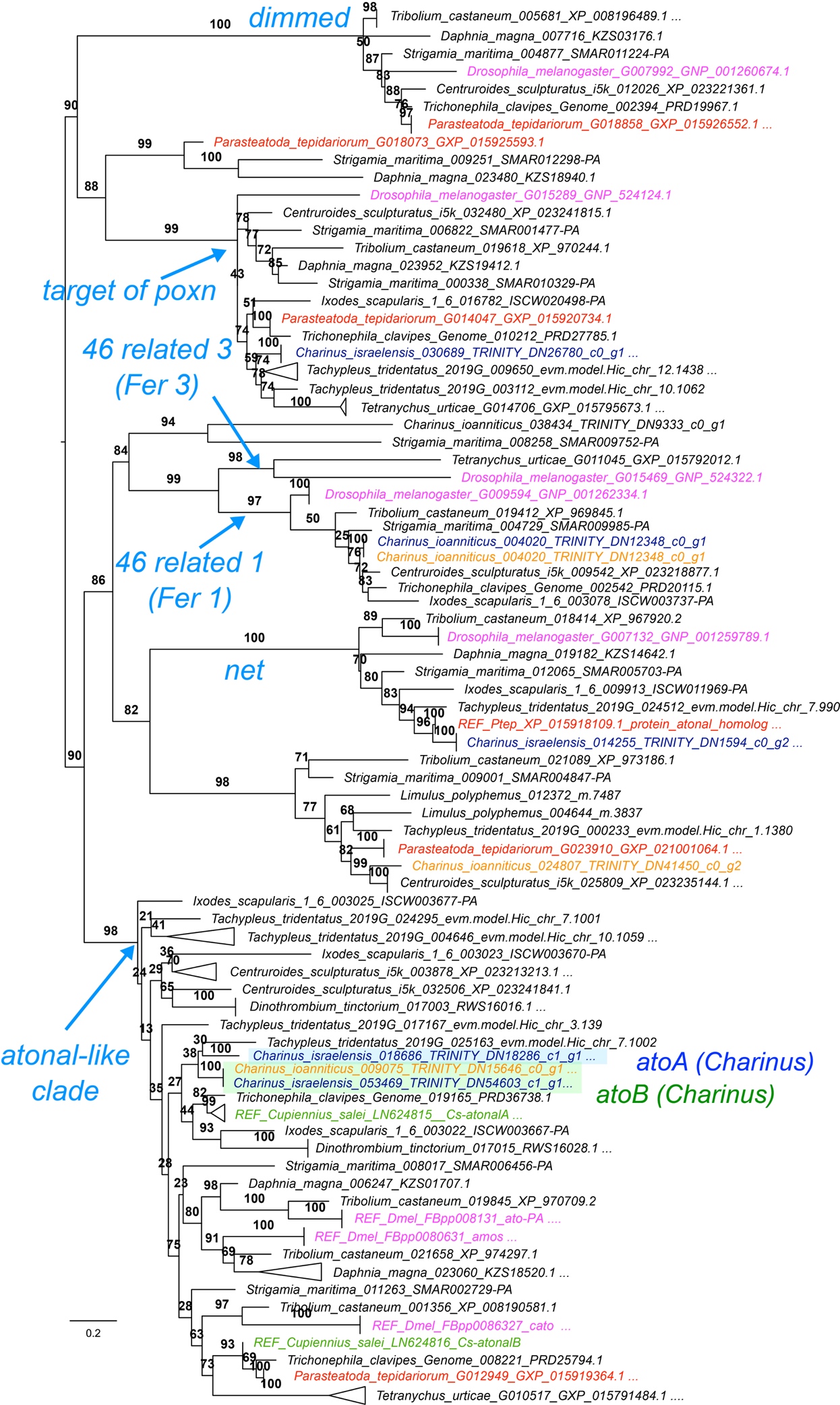
**

Additional file 1, Figure S2: Gene tree of atonal homologs. Multiple isoforms per gene were included for most terminals. Reference sequences of Drosophila melanogaster (cyan), Parasteatoda tepidariorum (red), and Cupiennius salei (green) were used to inform annotation (see Material and Methods). Terminals for Charinus ioanniticus and C. israelensis are colored orange and blue, respectively. Groups of inparalogous, alleles and isoforms were collapsed into an arbitrary representative sequence. Please refer to Newick tree files for details (Additional file 4, Dataset S1).

**
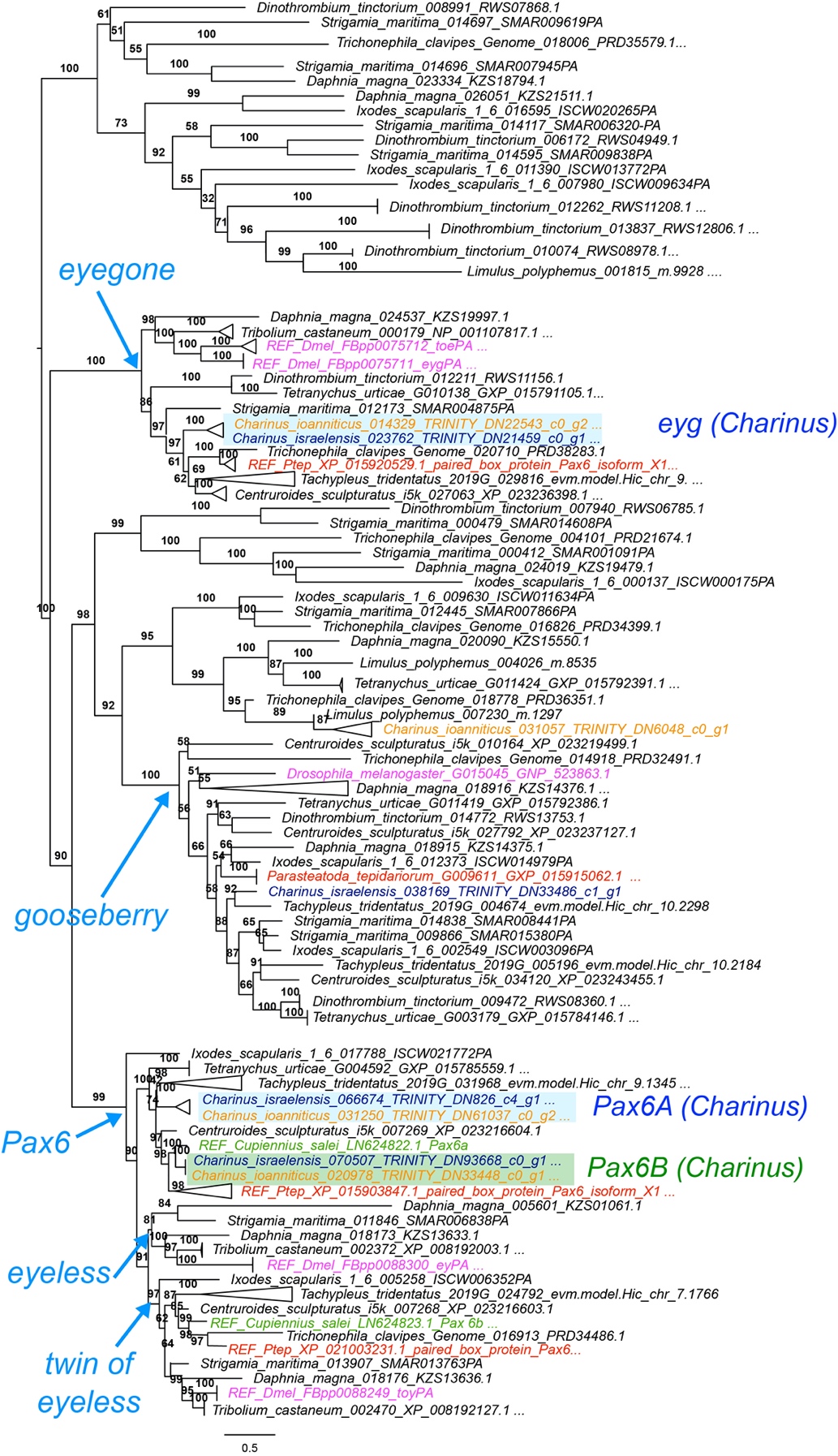
**

Additional file 1, Figure S3: Gene tree of Pax6 and eyegone homologs. Multiple isoforms per gene were included for most terminals. Reference sequences of Drosophila melanogaster (cyan), Parasteatoda tepidariorum (red), and Cupiennius salei (green) were used to inform annotation (see Material and Methods). Terminals for Charinus ioanniticus and C. israelensis are colored orange and blue, respectively. Groups of inparalogous, alleles and isoforms were collapsed into an arbitrary representative sequence. Please refer to Newick tree files for details (Additional file 4, Dataset S1).

**
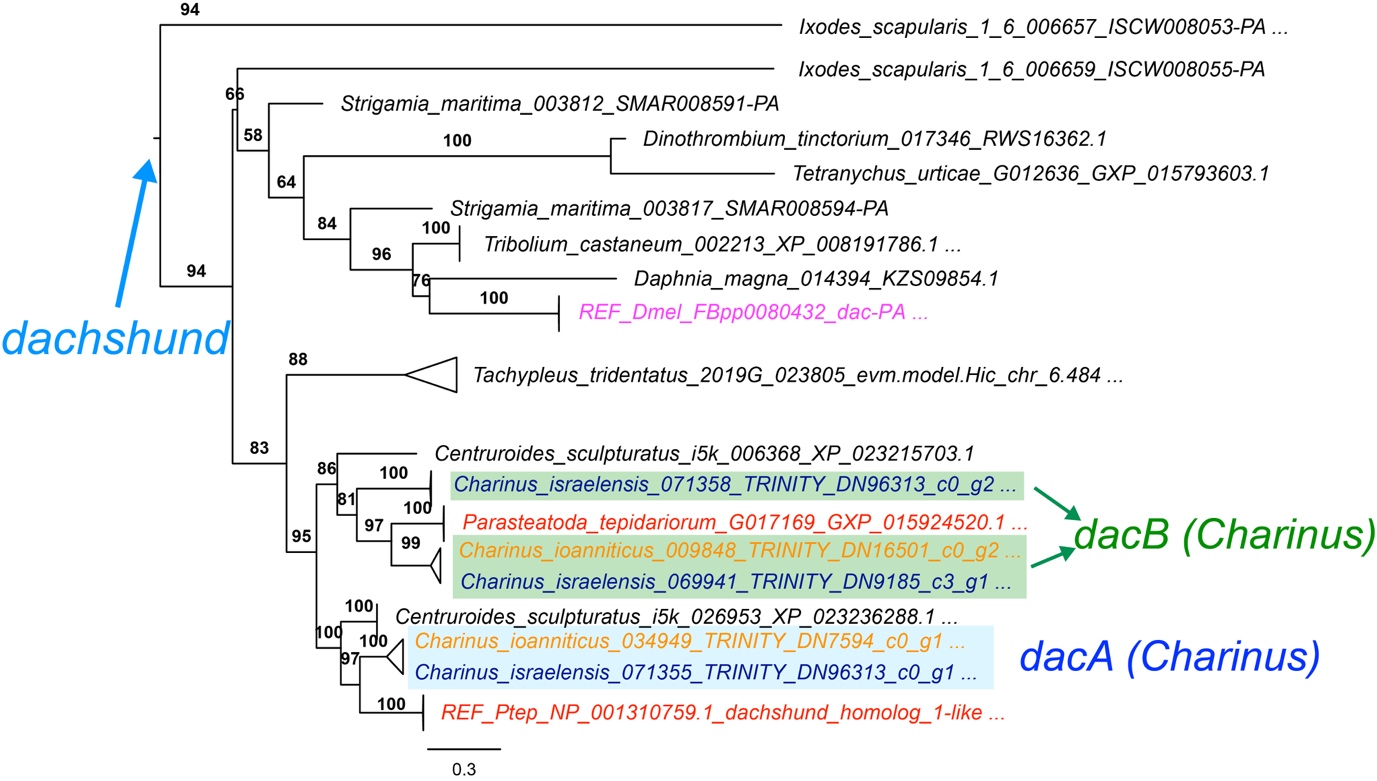
**

Additional file 1, Figure S4: Gene tree of dachshund homologs. Multiple isoforms per gene were included for most terminals. Reference sequences of Drosophila melanogaster (cyan) and Parasteatoda tepidariorum (red) were used to inform annotation (see Material and Methods). Terminals for Charinus ioanniticus and C. israelensis are colored orange and blue, respectively.The two well-defined clades of C. israelensis sequences in the dacB clade overlap in only three amino acids, and are probably two fragmentary assemblies of the same gene. Ixodes scapularis (Isca) has at least four dac copies which appear to be specific to this species. Groups of inparalogous, alleles and isoforms were collapsed into an arbitrary representative sequence. Please refer to Newick tree files for details (Additional file 4, Dataset S1).

**
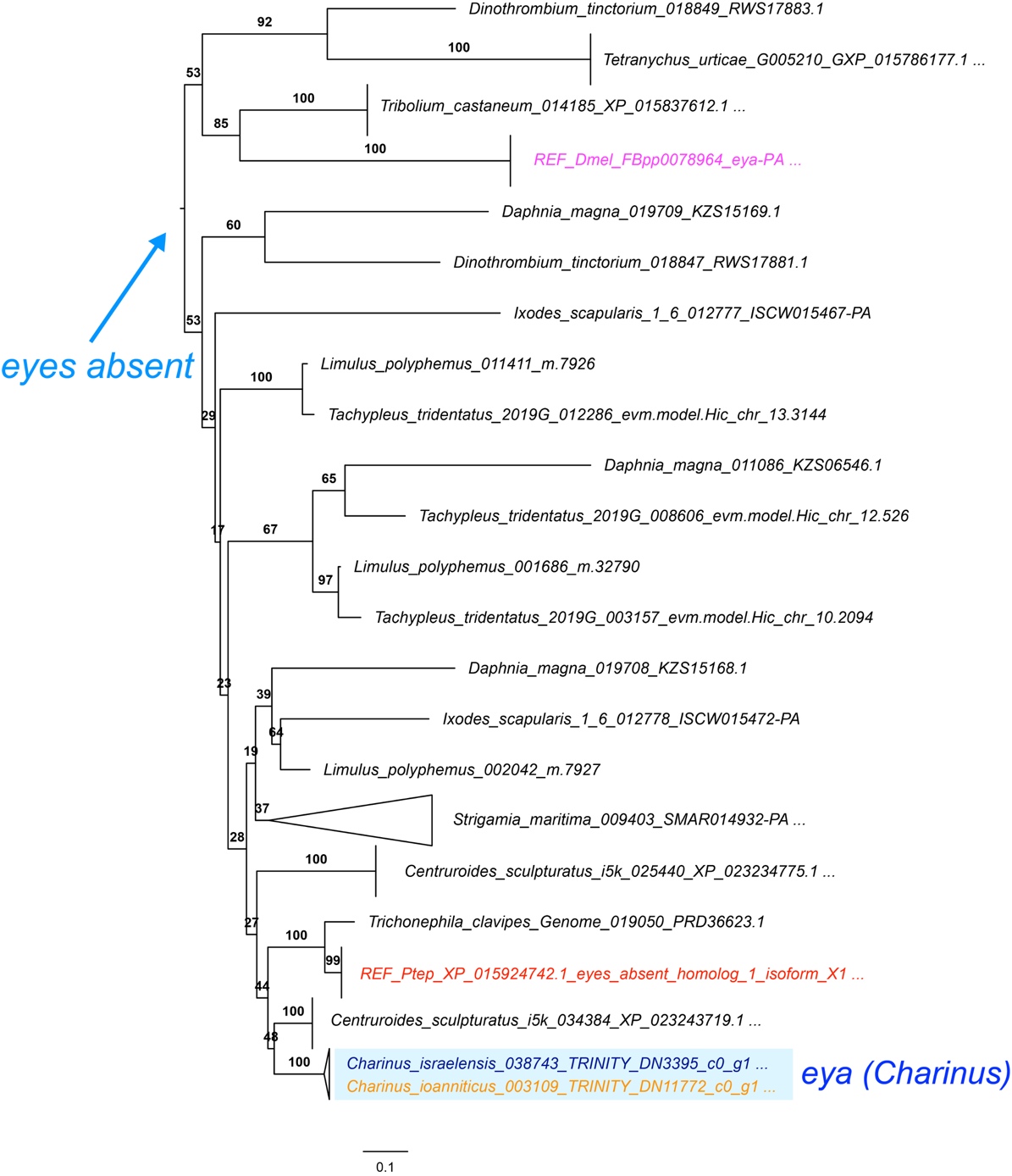
**

Additional file 1, Figure S5: Gene tree of eyes absent homologs. Multiple isoforms per gene were included for most terminals. Reference sequences of Drosophila melanogaster (cyan) and Parasteatoda tepidariorum (red) were used to inform annotation (see Material and Methods). Terminals for Charinus ioanniticus and C. israelensis are colored orange and blue, respectively. The terminals for Ixodes scapularis (Isca) and Dinothrombium tinctorium each appear to be fragmentary assemblies of a single copy, as they have little overlapping sequence between them (see alignment in Appendix Dataset S1). Groups of inparalogous, alleles and isoforms were collapsed into an arbitrary representative sequence. Please refer to Newick tree files for details (Additional file 4, Dataset S1).

**
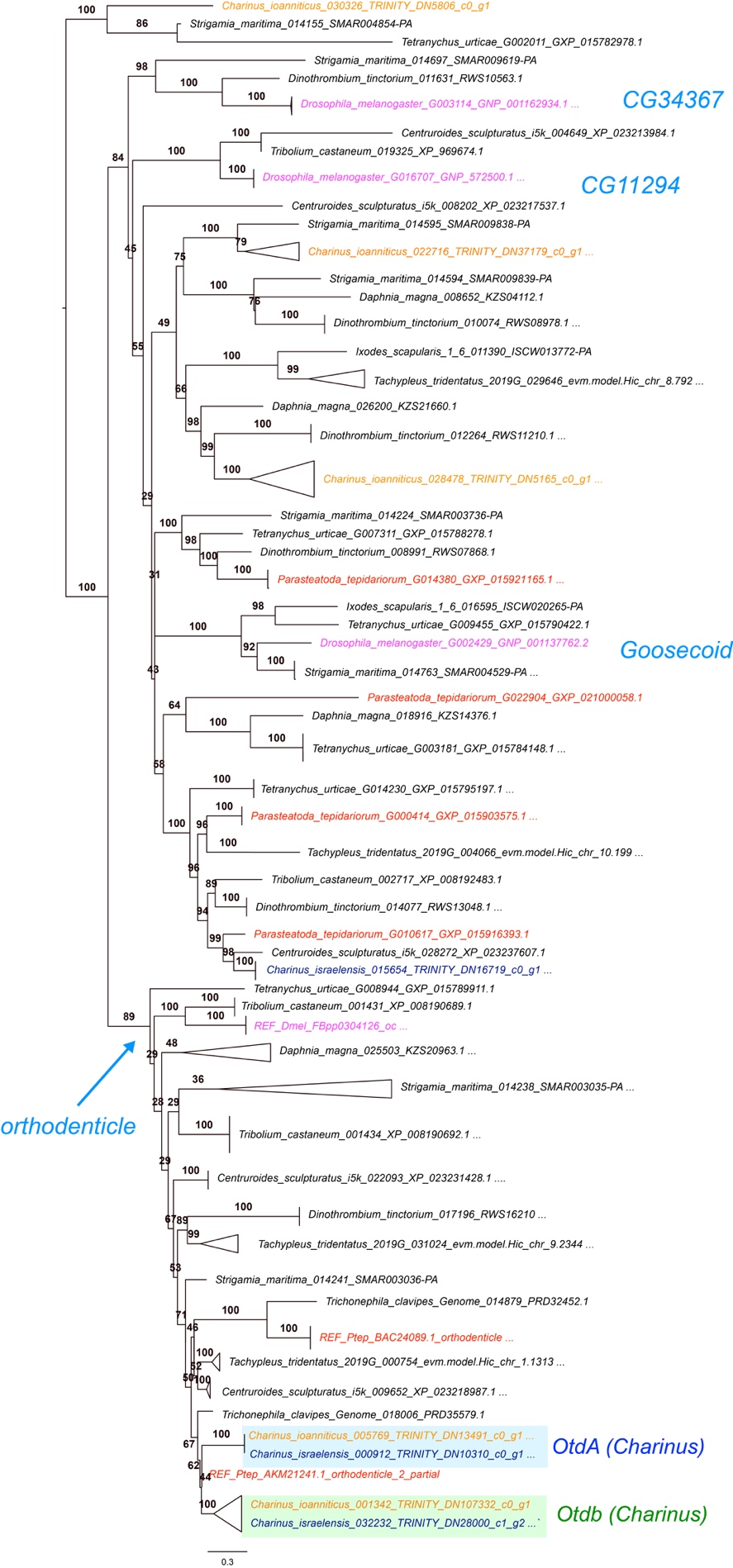
**

Additional file 1, Figure S6: Gene tree of goosecoid and orthodenticle homologs. Multiple isoforms per gene were included for most terminals. Reference sequences of Drosophila melanogaster (cyan) and Parasteatoda tepidariorum (red) were used to inform annotation (see Material and Methods). Terminals for Charinus ioanniticus and C. israelensis are colored orange and blue, respectively. Groups of inparalogous, alleles and isoforms were collapsed into an arbitrary representative sequence. Please refer to Newick tree files for details (Additional file 4, Dataset S1).

**
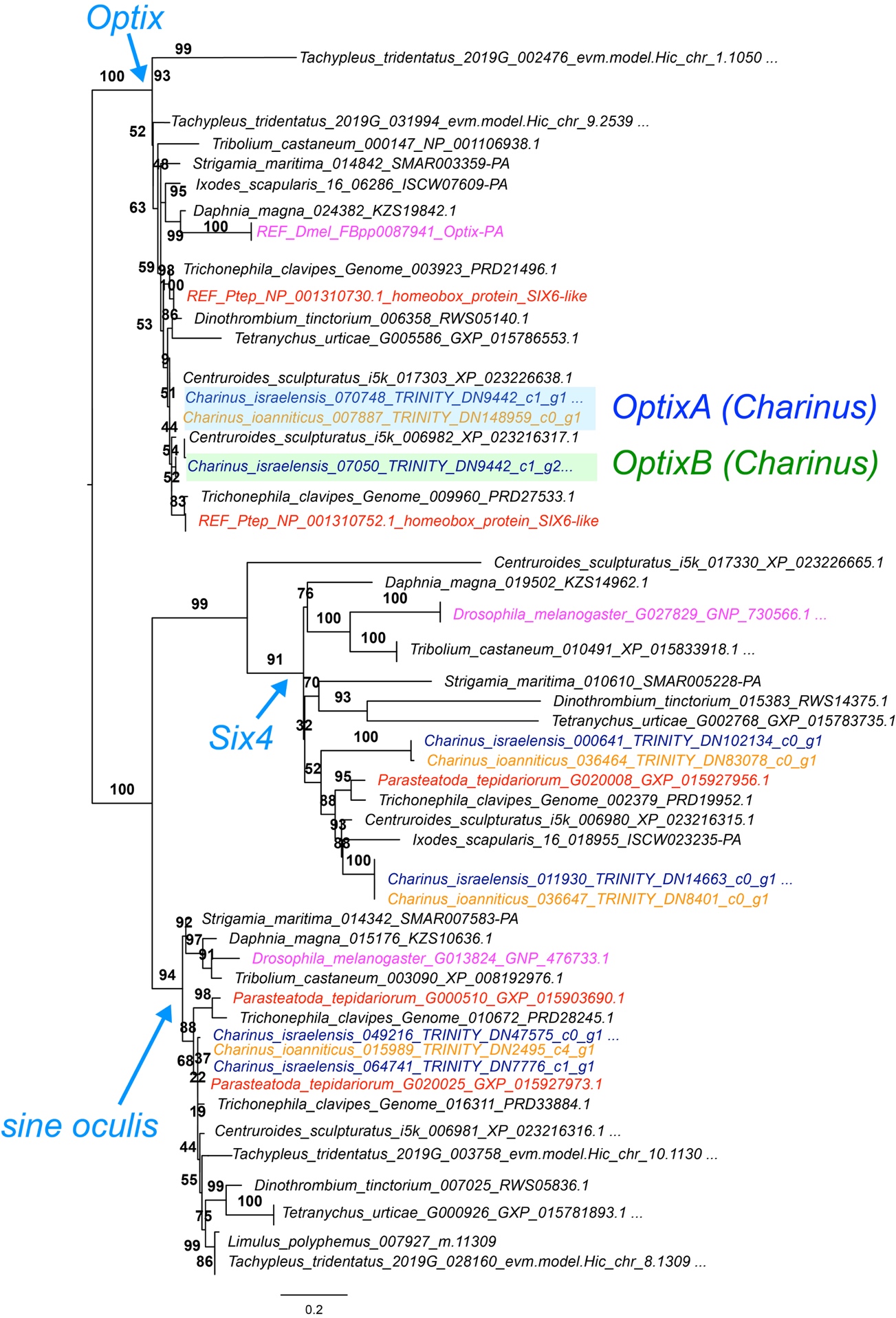
**

Additional file 1, Figure S7: Gene tree of Six genes homologs, including Six1 (sineoculis), Six3 (Optix) and Six4. Multiple isoforms per gene were included for most terminals. Reference sequences of Drosophila melanogaster (cyan) and Parasteatoda tepidariorum (red) were used to inform annotation (see Material and Methods). Terminals for Charinus ioanniticus and C. israelensis are colored orange and blue, respectively. Groups of inparalogous, alleles and isoforms were collapsed into an arbitrary representative sequence. Please refer to Newick tree files for details (Additional file 4, Dataset S1).

**
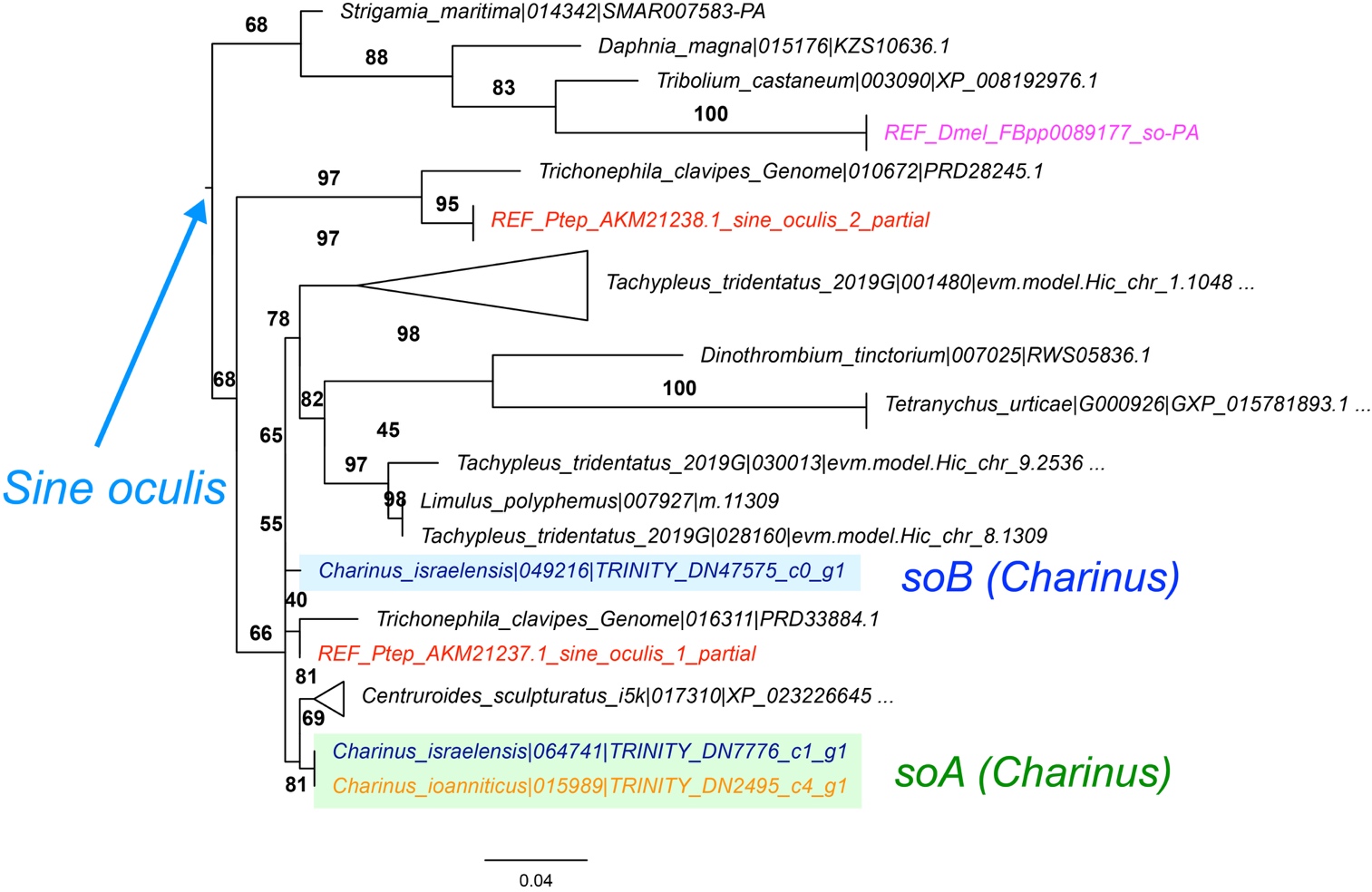
**
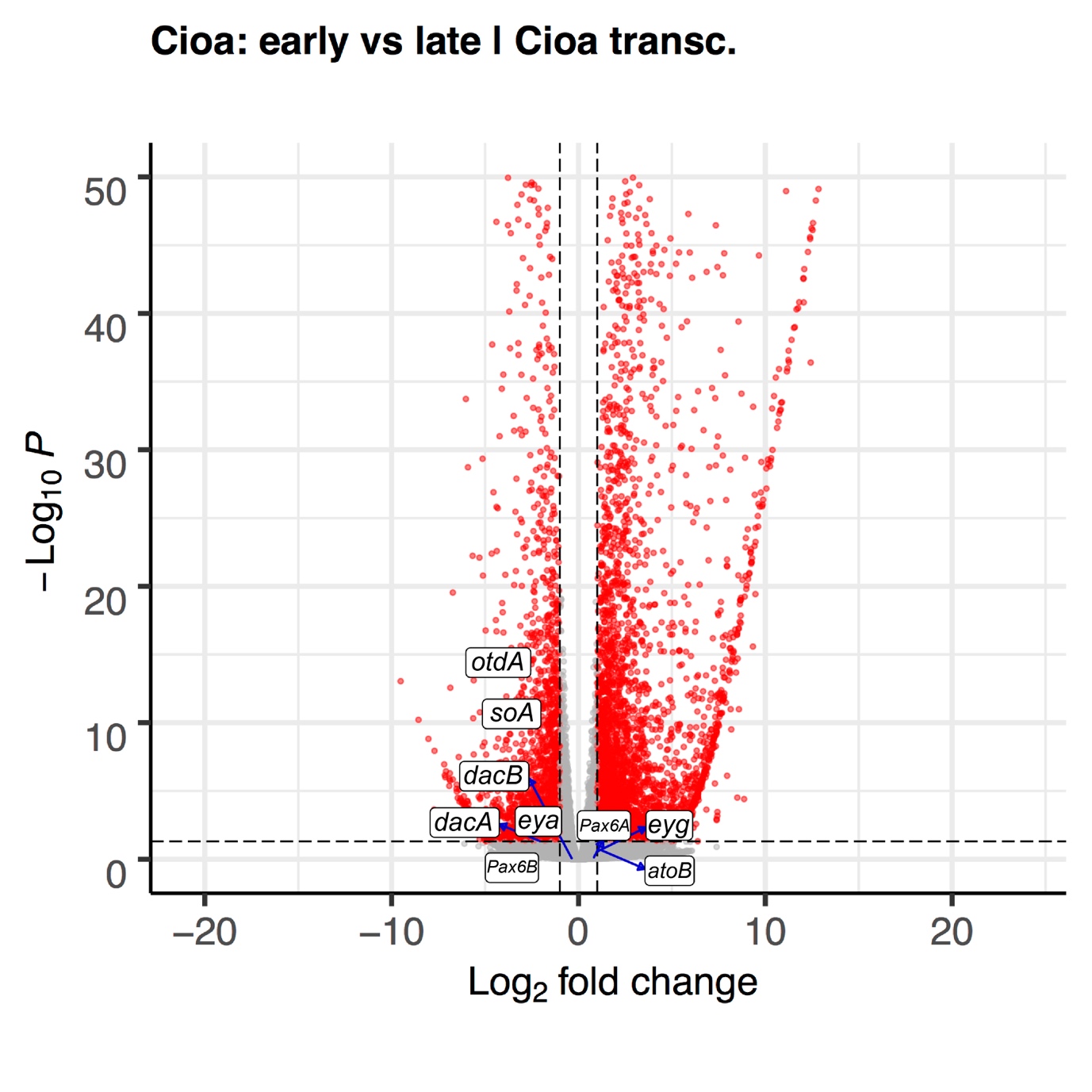


Additional file 1, Figure S8: Gene tree of sineoculis homologs. Multiple isoforms per gene were included for most terminals. Reference sequences of Drosophila melanogaster (cyan) and Parasteatoda tepidariorum (red) were used to inform annotation (see Material and Methods). Terminals for Charinus ioanniticus and C. israelensis are colored orange and blue, respectively. Groups of inparalogous, alleles and isoforms were collapsed into an arbitrary representative sequence. Please refer to Newick tree files for details (Additional file 4, Dataset S1).

Additional file 1, Figure S9: Comparison 1. Volcano plot of p_adj_ values (y-axis) and log_2_ fold change (x-axis) of all the genes in the analysis differential gene expression comparing reads from early deutembryos of *C. ioanniticus* (before eyespots) versus late deutembryos of *C. ioanniticus* (with eyespots). The denominator is the early deutembryo stage. Each gene is represented by a dot. Red dots have p_adj_>0.05. Dashed lines mark log_2_FC > [1].

**
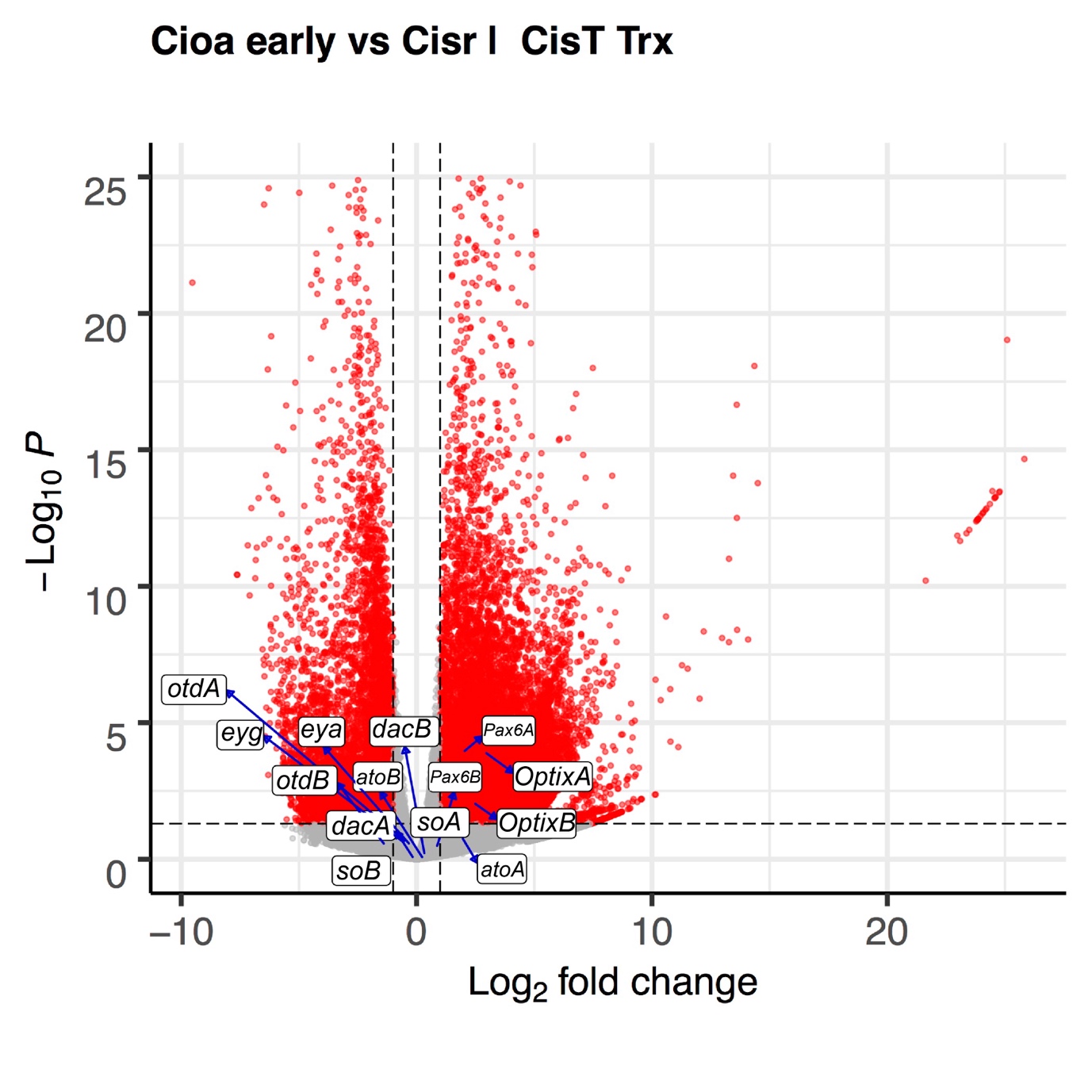
**

Additional file 1, Figure S10: Comparison 2.1. Volcano plot of p_adj_ values (y-axis) and log_2_ fold change (x-axis) of all the genes in the analysis differential gene expression comparing reads from early deutembryos of *C. ioanniticus* (normal-eyes) versus early deutembryos of *C. israelensis* (reduced-eyes) mapped onto *C. israelensis* transcriptome. The denominator is *C. ioanniticus*. Each gene is represented by a dot. Red dots have p_adj_>0.05. Dashed lines mark log_2_FC > [1].

**
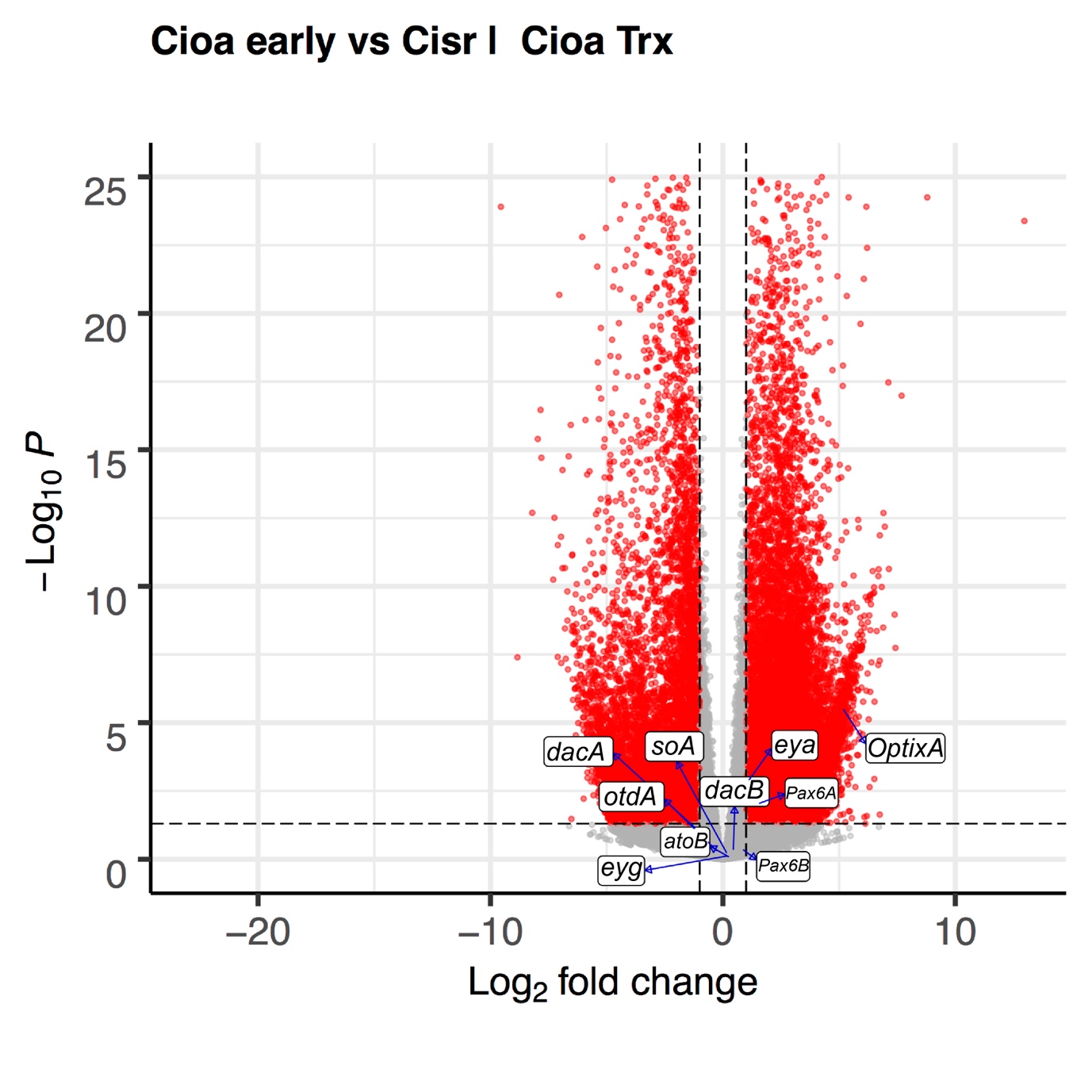
**

Additional file 1, Figure S11: Comparison 2.2. Volcano plot of p_adj_ values (y-axis) and log_2_ fold change (x-axis) of all the genes in the analysis differential gene expression comparing reads from early deutembryos of *C. ioanniticus* (normal-eyes) versus early deutembryos of *C. israelensis* (reduced-eyes) mapped onto *C. ioanniticus* transcriptome. The denominator is *C. ioanniticus*. Each gene is represented by a dot. Red dots have p_adj_>0.05. Dashed lines mark log_2_FC > [1].

**
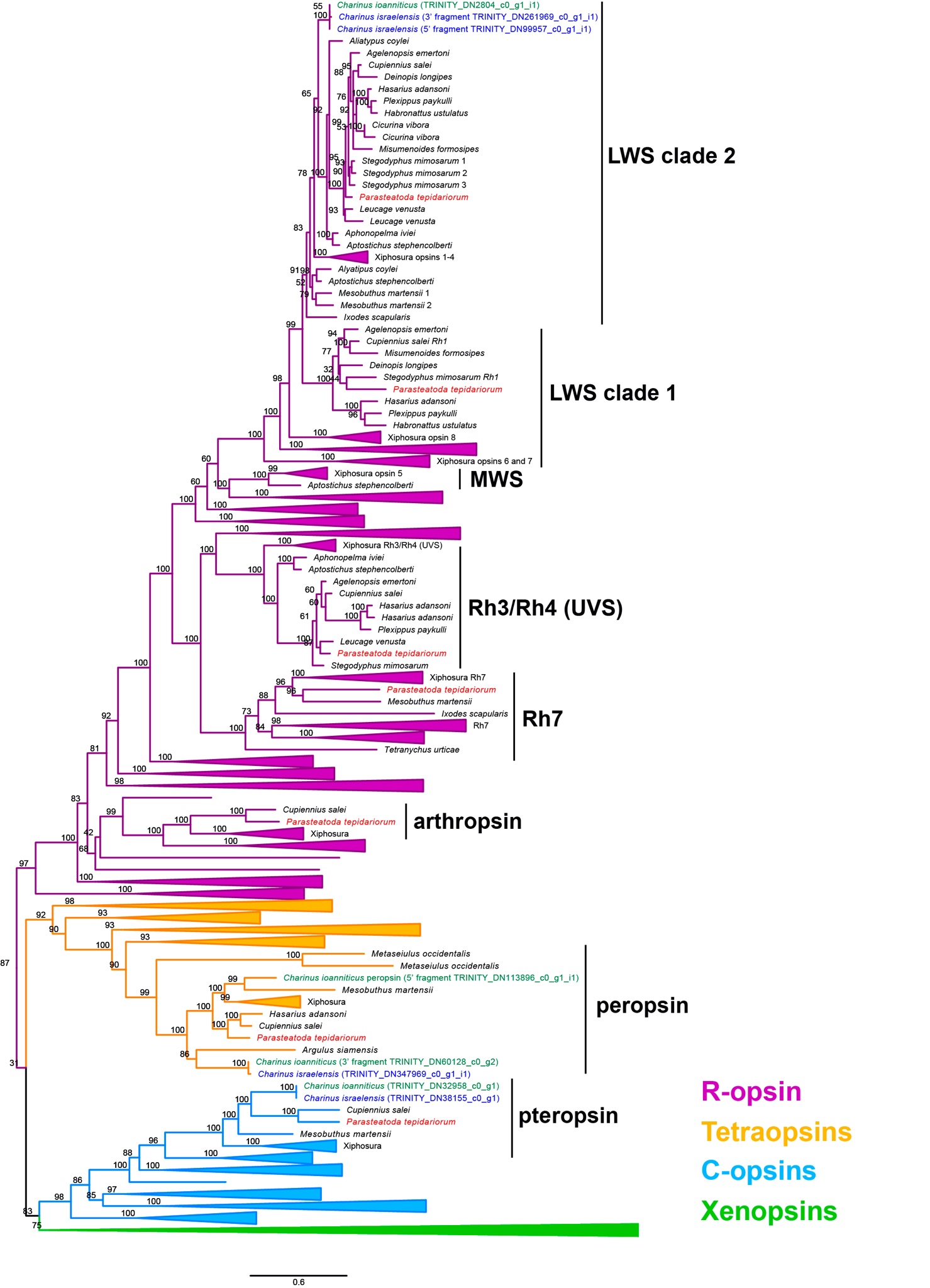
**

Additional file 1, Figure S12: Gene tree of metazoan opsins highlighting chelicerate sequences and the new sequences of *Charinus* whip spiders inferred from amino acid sequences. Original alignment and sequence IDs after [8,9]. The four main opsins lineages after [10] are color coded: Xenopsins (green), C-opsins (blue), Tetraopsins (ocre), and R-opsins/Gq-opsins (Cian). Specific arachnid opsins clades are denoted by vertical bars for long-wavelength-sensitive (LWS clade 1 and 2), middle-wavelength-sensitive (MWS), ultraviolet-sensitive (UVS Rh3 and Rh4), Rh7, arthropsin, peropsin, and pteropsin. Numbers on the nodes are ultra-fast bootstrap support values.


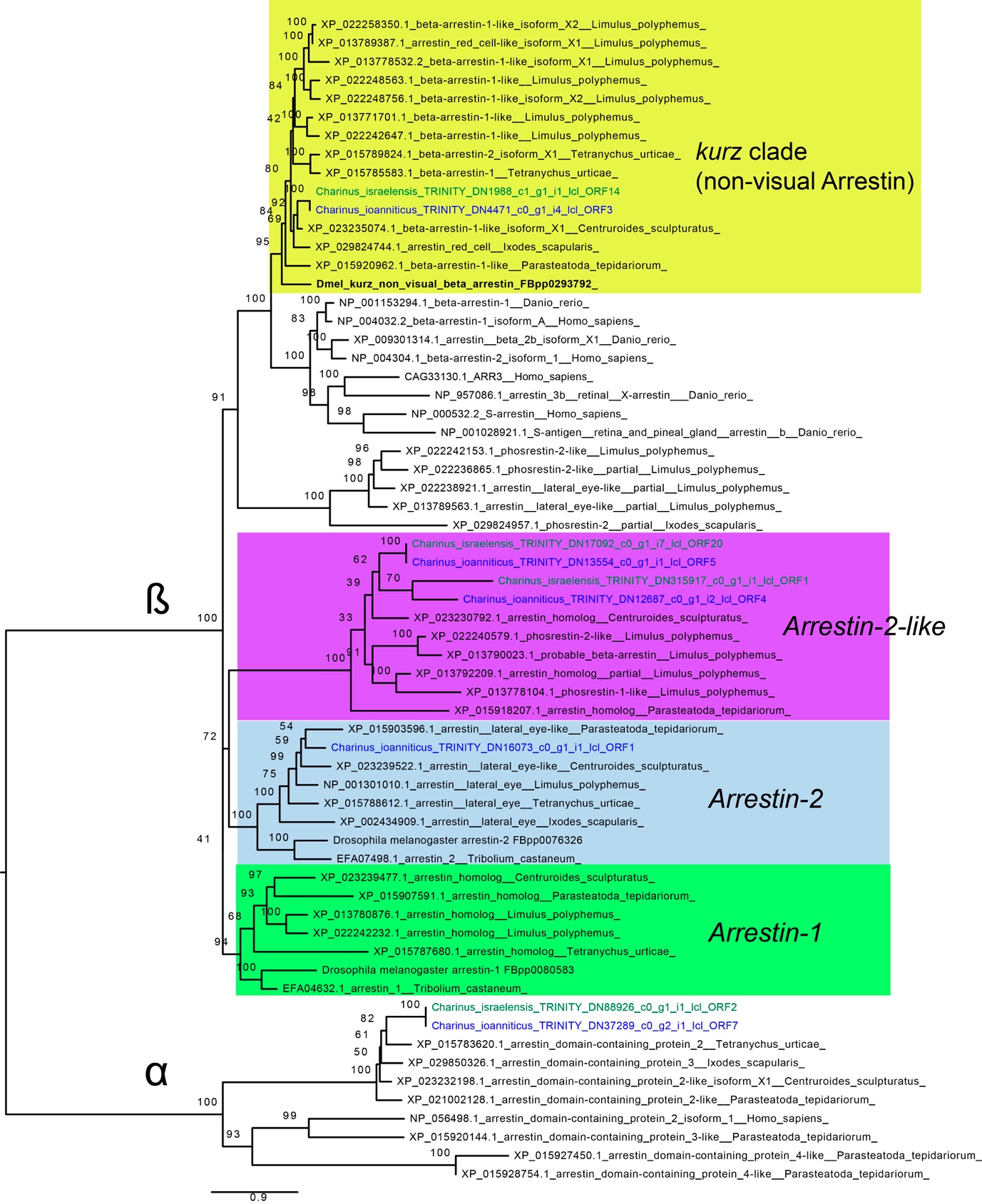


Additional file 1, Figure S13: Gene tree of arrestins inferred from amino acid sequences. Nomenclature follows [11]. Highlighted clades are *Arrestin-1* (green), *Arrestin-2* (blue). *Arrestin-2-like* (purple), and *kurtz* (yellow). Numbers on the nodes are ultra-fast bootstrap support values

**
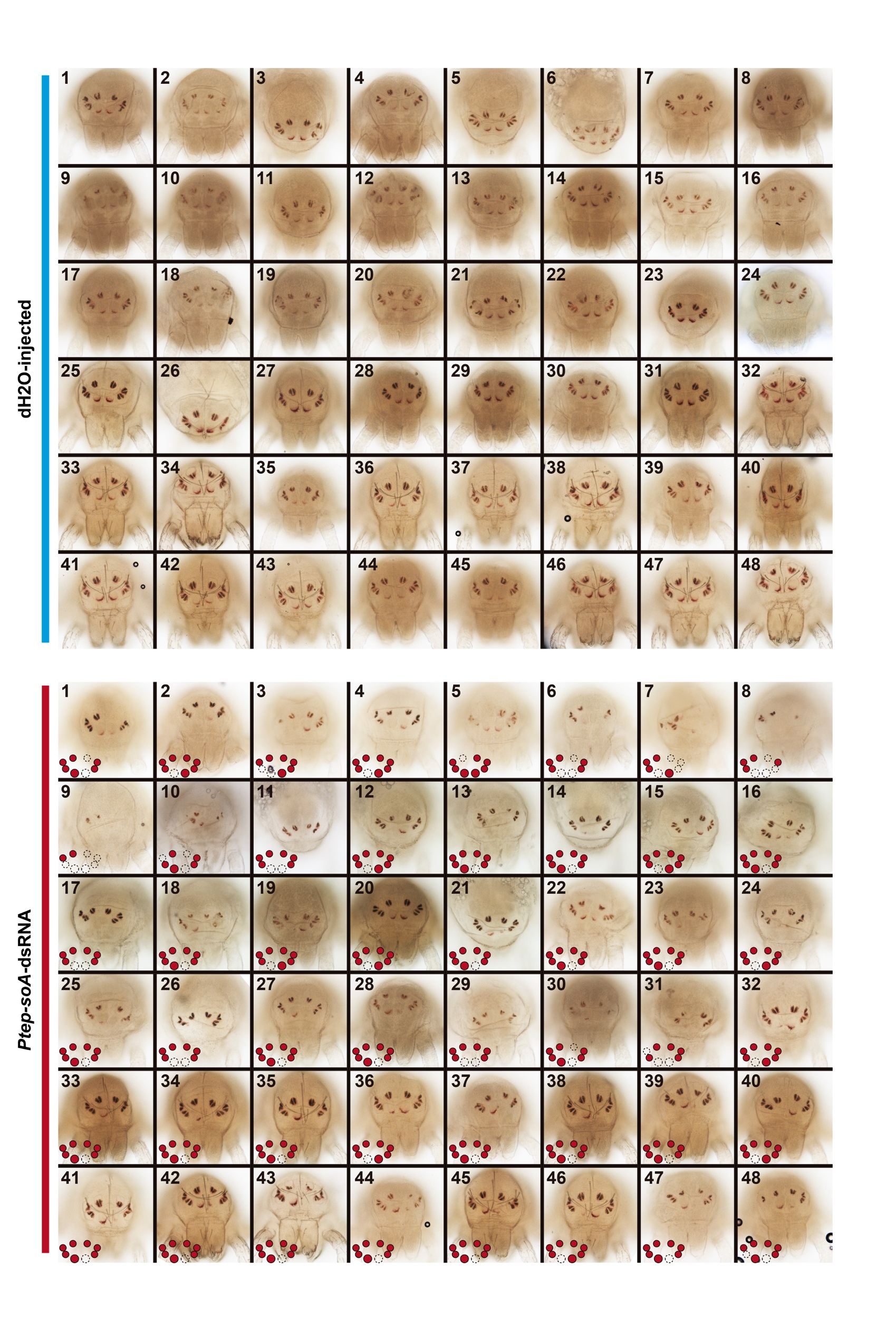
**

Additional file 1, Figure S14: Phenotypic spectrum of dH_2_O-injected (upper panel) and *Ptep-soA*-injected treatments used in the quantification of the effect per eye. High resolution image available at Additional file 6, Dataset S3.


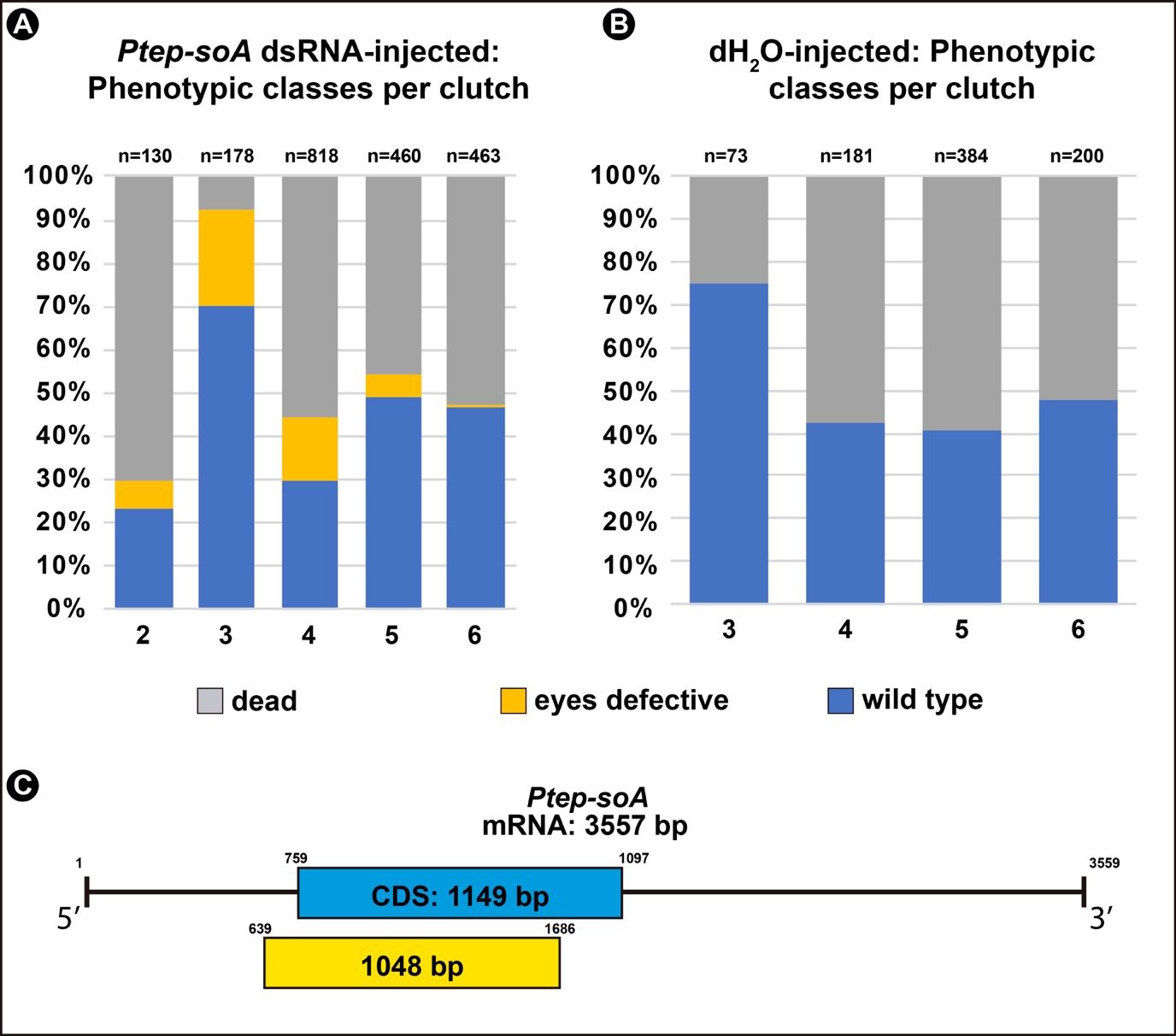


Additional file 1, Figure S15: A. Proportion of individuals in each phenotypic classes per clutch number of the *Ptep-soA* dsRNA-injected treatment. B. Proportions of individuals in each phenotypic classes per clutch number of the dH_2_O-injected treatment (control). Grey bars: dead; Yellow bar: eyes defective; Blue bar: wild type. C. Schematic representation of *Ptep-soA* transcript, with coding sequence (CDS) highlighted in blue. Yellow box represents the 1048 bp cloned fragment used for probe and dsRNA synthesis.

References:

1. Samadi L, Schmid A, Eriksson BJ. Differential expression of retinal determination genes in the principal and secondary eyes of *Cupiennius salei* Keyserling (1877). EvoDevo. BioMed Central; 2015;6:16–7.

2. Schwager EE, Sharma PP, Clarke T, Leite DJ, Wierschin T, Pechmann M, et al. The house spider genome reveals an ancient whole-genome duplication during arachnid evolution. BMC Biol. 2017;15:62.

3. Friedrich M. Ancient genetic redundancy of *eyeless* and *twin* of eyeless in the arthropod ocular segment. Developmental Biology. Elsevier Inc; 2017;432:192–200.

4. Schomburg C, Turetzek N, Schacht MI, Schneider J, Kirfel P, Prpic N-M, et al. Molecular characterization and embryonic origin of the eyes in the common house spider *Parasteatoda tepidariorum*. EvoDevo. BioMed Central; 2015;6:15.

5. Turetzek N, Pechmann M, Schomburg C, Schneider J, Prpic N-M. Neofunctionalization of a Duplicate *dachshund* Gene Underlies the Evolution of a Novel Leg Segment in Arachnids. Mol. Biol. Evol. 2015;33:109–21.

6. Nolan ED, Santibáñez López CE, Sharma PP. Developmental gene expression as a phylogenetic data class: support for the monophyly of Arachnopulmonata. Dev. Genes Evol. Springer Berlin Heidelberg; 2020;230:137–53.

7. Mahato S, Morita S, Tucker AE, Liang X, Jackowska M, Friedrich M, et al. Common Transcriptional Mechanisms for Visual Photoreceptor Cell Differentiation among Pancrustaceans. Desplan C, editor. PLoS Genet. 2014;10:e1004484–15.

8. Morehouse NI, Buschbeck EK, Zurek DB, Steck M, Porter ML. Molecular Evolution of Spider Vision: New Opportunities, Familiar Players. Biol Bull. 2017;233:21–38.

9. Battelle B-A, Ryan JF, Kempler KE, Saraf SR, Marten CE, Warren WC, et al. Opsin Repertoire and Expression Patterns in Horseshoe Crabs: Evidence from the Genome of *Limulus polyphemus* (Arthropoda: Chelicerata). Genome Biol Evol. 2016;8:1571–89.

10. Ramirez MD, Pairett AN, Pankey MS, Serb JM, Speiser DI, Swafford AJ, et al. The last common ancestor of most bilaterian animals possessed at least 9 opsins. Genome Biol Evol. 2016;:evw248–13.

11. Alvarez CE. On the origins of arrestin and rhodopsin. BMC Evol Biol. BioMed Central; 2008;8:222–13.

Additional file 1, Table S1: Collecting details and sample description for all sequenced samples and respective vouchers.

| **Taxonomy** | **Collecting date** | **ID** | **Locality** | **Longitude** | **Latitude** | **Elevation (m)** | **Preservation** | **Use** | **Description** |
| --- | --- | --- | --- | --- | --- | --- | --- | --- | --- |
| *Charinus ioanniticus* | 7/19/18 | ISR021-2 | Hirbet Haruba Cave, deeper chamber | 34.96083 | 31.91328 | 189 | RNAlater | DGE | embryos of female 2 (pre-eye stage); n = 10 |
| *Charinus ioanniticus* | 7/19/18 | ISR021-3 | Hirbet Haruba Cave, deeper chamber | 34.96083 | 31.91328 | 189 | RNAlater | transcriptome assembly; DGE | embryos of female 3 (pre-eye stage); n = 13 |
| *Charinus ioanniticus* | 7/19/18 | ISR021-4 | Hirbet Haruba Cave, deeper chamber | 34.96083 | 31.91328 | 189 | RNAlater | transcriptome assembly; DGE | embryos of female 4 (eye stage); n = 13 |
| *Charinus ioanniticus* | 7/19/18 | ISR021-5 | Hirbet Haruba Cave, deeper chamber | 34.96083 | 31.91328 | 189 | FA/PBST | voucher morphology | embryos of female 2 (pre-eye stage); n = 2 |
| *Charinus ioanniticus* | 7/19/18 | ISR021-6 | Hirbet Haruba Cave, deeper chamber | 34.96083 | 31.91328 | 189 | FA/PBST | voucher morphology | embryos of female 3 (pre-eye stage); n = 2 |
| *Charinus ioanniticus* | 7/19/18 | ISR021-7 | Hirbet Haruba Cave, deeper chamber | 34.96083 | 31.91328 | 189 | FA/PBST | voucher morphology | embryos of female 4 (eye stage); n = 13 |
| *Charinus israelensis* | 7/27/18 | ISR051-4 | Cistern inside Mimlach Cave | 35.44411 | 32.85815 | 139 | RNAlater | transcriptome assembly | embryos of female 2 (deutembryo); n=5 |
| *Charinus israelensis* | 7/27/18 | ISR051-5 | Cistern inside Mimlach Cave | 35.44411 | 32.85815 | 139 | FA/PBST | voucher morphology | embryos of female 2 (deutembryo); n=2 |
| *Charinus israelensis* | 7/27/18 | ISR051-6 | Cistern inside Mimlach Cave | 35.44411 | 32.85815 | 139 | RNAlater | transcriptome assembly; DGE | embryos of female 3 (deutembryo); n=10, |
| *Charinus israelensis* | 7/27/18 | ISR051-7 | Cistern inside Mimlach Cave | 35.44411 | 32.85815 | 139 | FA/PBST | voucher morphology | embryos of female 3 (deutembryo); n=2 |

| **Species** | **Total trinity genes** | **Total trinity transcripts** | **% GC** | **Median contig length** | **Mean contig length** | **Total assembled bases** | **Contig N50 (all transcripts)** | **Median contig length (longest gene)** | **Average contig (longest gene)** | **Total assembled bases (longest)** | **BUSCO** |
| --- | --- | --- | --- | --- | --- | --- | --- | --- | --- | --- | --- |
| *Charinus ioanniticus* | 170848 | 219797 | 39.23 | 332 | 651.88 | 143282365 | 1122 | 305 | 552.92 | 94466089 | C:93.8%[S:88.1%,D:5.7%],F:3.8%,M:2.4%,n:1066 |
| *Charinus israelensis* | 477982 | 663281 | 38.58 | 323 | 630.61 | 418268343 | 1045 | 297 | 481.28 | 230044656 | C:95.2%[S:88.4%,D:6.8%],F:2.5%,M:2.3%,n:1066 |

Additional file 1, Table S2: Summary statistics of transcriptomes of *Charinus ioanniticus* and *Charinus israelesis*.
