# Supplementary figures and images for: Systemic paralogy and function of retinal determination network homologs in arachnids
Source: BMC Genomics. 2020 Nov 23;21:811. doi: 10.1186/s12864-020-07149-x (PMC7681978; doi:10.1186/s12864-020-07149-x)

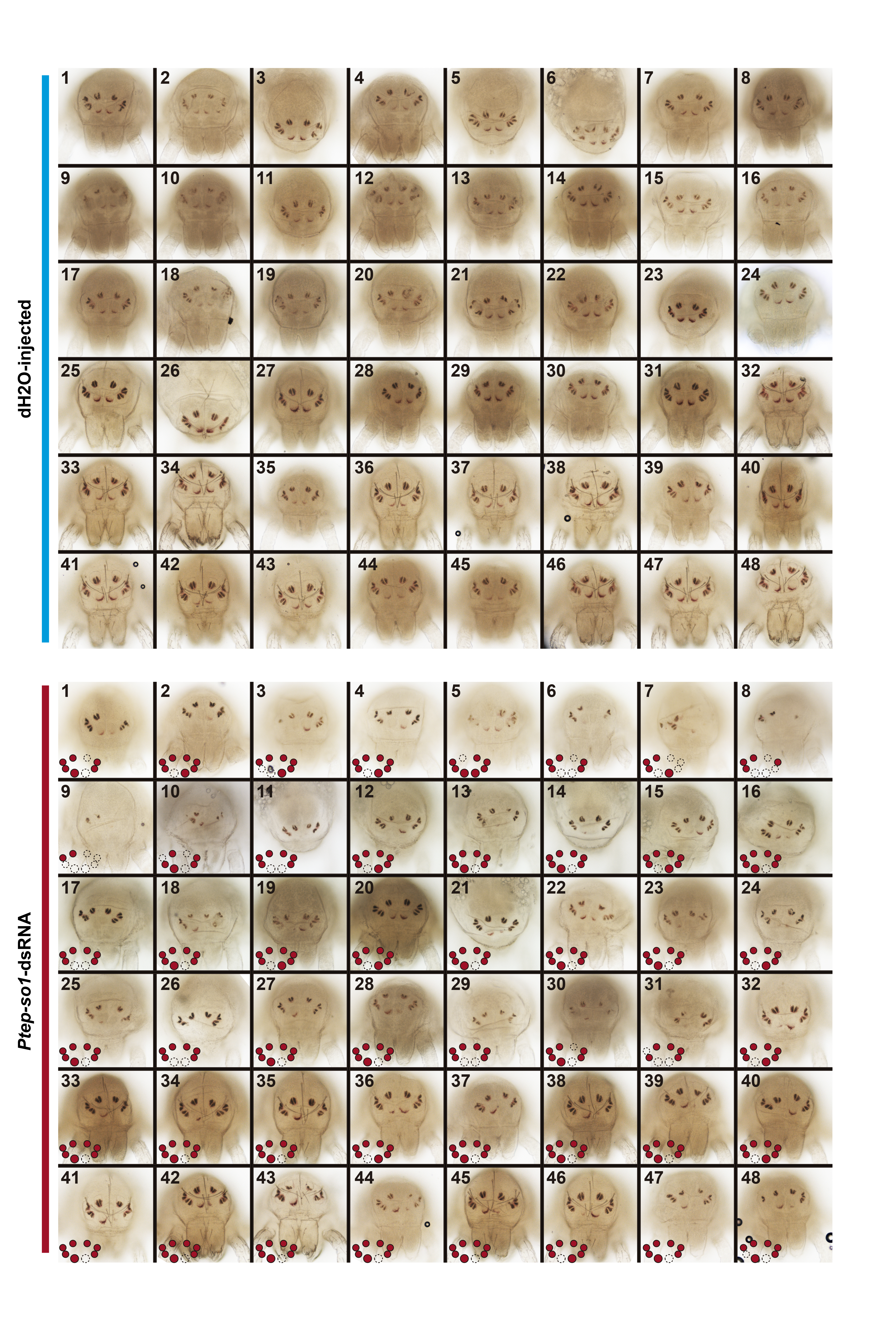

Supplement: Supplementary file 7 — Additional file 7. Dataset S4. (a): High resolution Additional file 1 Fig. S14. (b): Spreadsheets with the raw counts and sum of counts used to generate the distribution bar plots of the Ptep-soA RNAi experiment. (c) raw counts and sum of counts used to generate the distribution bar plots of the effects of Ptep-soA RNAi per eye type. (d) Primer sequences for the amplified fragments of Ptep-soA, Ptep-otdB and Ptep-OptixB. (.zip). Available at: https://datadryad.org/stash/share/xb2VW4o80AmId3mLFZB07Ho7rHxPx-htK9q5J_-2miM; doi:https://doi.org/10.5061/dryad.xgxd254d1 [file 12864_2020_7149_MOESM7_ESM.zip › Additional_file7_Dataset_S4_RNAi_results/Dataset_S4_A_Fig_S15.jpg]
